# Multi-omics network inference with a Gaussian copula model

**DOI:** 10.1186/s12859-026-06502-3

**Published:** 2026-06-04

**Authors:** Ekaterina Tomilina, Gildas Mazo, Florence Jaffrézic

**Affiliations:** 1 Université Paris-Saclay, INRAE, MaIAGE, 78350 Jouy-en-Josas, France; 2 Université Paris-Saclay, INRAE, AgroParisTech, GABI, 78350 Jouy-en-Josas, France

**Keywords:** Partial correlation networks, Multi-omics data, Gaussian copula, Graphical lasso

## Abstract

**Background:**

Inferring partial correlation networks is essential in systems biology to uncover direct interactions between biological entities. Traditional Gaussian graphical models rely on the assumption of normally distributed data; this assumption is not satisfied when dealing with multi-omics datasets comprising heterogeneous data types such as continuous and discrete variables.

**Results:**

We propose a novel likelihood-based approach for network inference using a Gaussian copula model with semiparametric pairwise-likelihood estimation of the latent correlation matrix. The inferred correlation structure is then inverted and regularized via the graphical lasso to recover latent partial correlations. Compared to a moment-based approach employing bridge functions, our method demonstrates significantly improved computational efficiency and estimation accuracy, particularly for discrete data with many categories and/or large values, such as count data. This result is important for biological applications, especially for the integration of RNA-seq count data. An application to a breast cancer data set from the International Cancer Genome Consortium (ICGC) successfully identified biologically relevant interactions.

**Conclusions:**

The proposed approach, based on the Gaussian copula and likelihood-based estimation, provides a novel, effective and computationally efficient mathematical framework for integrative multi-omics data analysis and network inference.

## Introduction

Systems biology is based on the analysis of complex, large-scale data of diverse nature. A key biological challenge is to understand the interactions and regulatory links between the different types of omics data. Their heterogeneity constitutes a major analysis issue. Some can easily be modeled with Gaussian distributions (transcriptomic data from microarrays), others are continuous but non-Gaussian (epigenomic data from methylation chips), or discrete (transcriptomic and epigenomic data from sequencing, genotyping). However, mathematical and statistical models are highly dependent on the nature of the data, which is why the models proposed to date have mainly been carried out independently for each omics level.

When the data are Gaussian, the networks are assimilated to a set of conditional independence relationships between variables. These relationships are encoded in the inverse of the covariance matrix, called the precision matrix, of the underlying Gaussian distribution. In this case, inference is made by penalized maximum likelihood. A commonly used algorithm for that purpose is called glasso [1]. When the data are continuous, but not necessarily Gaussian, a non-linear scaling of the data allows to recover Gaussian data [2]. This approach has been used, for example, to analyze microarray data. When the data are discrete, like the counts generated by RNA-seq technology, there is no one-to-one transformation to recover Gaussian data. An alternative is to build a hierarchical model, such as Poisson models based on latent Gaussian variables [3]. The network is reconstructed from the precision matrix of latent variables. This approach has the advantage of not requiring any transformation, but is limited to the analysis of one type of data with strong parametric assumptions regarding the observed distribution.

In the context of multi-omics network inference, it is necessary to be able to study the relationships between data of various types (Gaussian, continuous non-Gaussian, counts, etc.) Two main approaches have been proposed to tackle this issue [4]. The first one corresponds to Mixed Graphical Models, which are an extension of Gaussian graphical models allowing to take into account links between discrete and continuous variables by regressing each variable with respect to the others. It corresponds to an extension of the graphical lasso, with one or more regularization parameters [5, 6]. However, they require strong parametric assumptions on the marginal distributions. The second one corresponds to Gaussian copula-based graphical models. They assume that the observed variables correspond to marginal transformations of a latent Gaussian structure. Thus, these models are particularly well-suited for the integration of data with various types and can be extended to the semi-parametric case where only the copula parameter is to be estimated, with no assumptions regarding the marginals. Parameter inference for these models has so far been mostly performed in a Bayesian framework by MCMC approaches [7–9], which have a high computational cost. Some methods also rely on bridge functions that link an extension of Kendall’s tau (estimated on the observed variables) to the copula correlation coefficient [10]. The latter is then estimated by solving the corresponding equation, but it does not always have a solution on the considered parameter space.

The goal of this article is to propose a parameter inference algorithm based on the pairwise likelihood. We estimate the copula parameters directly from the observed data before applying the graphical Lasso in order to invert and regularize the inferred correlation matrix. The proposed algorithm is evaluated in an extensive simulation study in which we compare our performance to bridge function methods, and then applied to real biological data from the International Cancer Genome Consortium [11]. The method is implemented in an R package called heterocop, available on the CRAN.

## Methods

### Gaussian copula model

Let $X^i=(X_1^i, \dots , X_d^i)$, *i=1, … , n*, be a sample of i.i.d. observations in $\mathbb {R}^d$, and let *F* denote the cumulative distribution function (CDF) of $X^i$. We assume

1 $$\begin{aligned} \begin{aligned} F(x_1,..., x_d)&=C_{\Sigma }(F_1(x_1), \dots , F_d(x_d))\\&\equiv \Phi _{\Sigma }(\Phi ^{-1}(F_1(x_1)),..., \Phi ^{-1}(F_d(x_d))) \end{aligned} \end{aligned}$$

where $F_1, \dots , F_d$ denote the marginal CDFs of $X_1^i, \dots X^i_d$, $\Phi _{\Sigma }$ the centered Gaussian multivariate CDF of correlation matrix *Σ *, and $\Phi ^{-1}$ the inverse standard Normal CDF.

In other words, model (1) corresponds to a latent Gaussian variable structure. Let $Z^i=(Z^i_1,...., Z^i_d)\sim {\mathcal {N}}(0, \Sigma )$ denote a centered standardized Gaussian vector of correlation matrix *Σ *. Let $F^{\leftarrow }_j$ denote the generalized inverse function such that $F^{\leftarrow }_j(u)=\inf \{x: F_j(x) \ge u \}$. If $X^i_j=F_j^\leftarrow (\Phi (Z^i_j))$, then it can be shown that $X^i$ has the CDF given by (1) (see Sect. D of the Supplementary Material and [34]).

We are interested in the estimation of the partial covariances between the latent Gaussian variables $Z^i$,

2 $$\begin{aligned} Cov(Z^i_j,Z^i_{j'}|Z^i_{-(j,j') })=\mathbb {E}(Z^i_jZ^i_{j'}|Z^i_{-(j,j')})-\mathbb {E}(Z^i_j|Z^i_{-(j,j')})\mathbb {E}(Z^i_{j'}|Z^i_{-(j,j')}), \end{aligned}$$

where $Z^i_{-(j,j')}$ denotes the set of variables $Z^i_1, \dots , Z^i_d$ without $Z^i_j$ and $Z^i_{j'}$. Note that for a given pair of variables $Z^i_j$ and $Z^i_{j'}$, this value is deterministic because it does not depend on the values taken by $Z^i_{-(j,j')}$. Indeed, it only depends on the values of *Σ * and is given by [12]: $Cov(Z^i_j,Z^i_{j'}|Z^i_{-(j,j') })=\Sigma _{jj'}-\Sigma _{\{(j,j'),-(j,j')\}}[\Sigma _{-(j,j')}]^{-1}\Sigma _{\{-(j,j'),(j,j')\}}$. It can be shown that

$$\begin{aligned} Cor(Z^i_j,Z^i_{j'}|Z^i_{-(j,j')})= & \dfrac{Cov(Z^i_j,Z^i_{j'}|Z^i_{-(j,j')})}{\sqrt{Var(Z^i_j|Z^i_{-(j,j')})Var(Z^i_{j'}|Z^i_{-(j,j')})}} \\ = & \dfrac{-\Omega _{jj'}}{\sqrt{\Omega _{jj}}\sqrt{\Omega _{j'j'}}}, \end{aligned}$$

where $\Omega _{jj'}$ denotes the element at the *j*-th row and *j'*-th column of the precision matrix $\Omega =\Sigma ^{-1}$.

### Pairwise-likelihood and Graphical Lasso

The first step is to obtain an estimation of matrix *Σ *. We propose to use the pairwise pseudo maximum likelihood estimator (PPMLE) studied by Mazo et al. [13], extended to the case of non-parametric marginals [14]:

3 $$\begin{aligned} {\hat{\Sigma }}=\underset{\Sigma }{\text {argmax}}\left( \dfrac{1}{n}\sum _{i=1}^n \sum _{j < j'} \log {\hat{f}}_{jj'}(X_j^i,X_{j'}^i,\Sigma _{jj'}) \right) \end{aligned}$$

where ${\hat{f}}_{jj'}(\cdot ,\cdot ,\Sigma _{jj'}) = f_{jj'}(\cdot ,\cdot ,{\hat{F}}_j,{\hat{F}}_{j'},\Sigma _{jj'})$ denotes an estimate of $f_{jj'}(\cdot ,\cdot ,F_j,F_{j'},\Sigma _{jj'})$, the density of the bivariate marginal CDF from model (1) corresponding to the pair $(X_j, X_{j'})$ with respect to the $\lambda \otimes \lambda$ measure if both variables are continuous, $\mu \otimes \mu$ measure if both variables are discrete, and $\lambda \otimes \mu$ measure if $X_j$ is continuous and $X_{j'}$ is discrete. *λ * denotes the Lebesgue measure and *μ * denotes the counting measure. Note that the marginal CDFs $F_j$ and $F_{j'}$ have been replaced by their empirical counterparts ${\hat{F}}_j$ and ${\hat{F}}_{j'}$, where ${\hat{F}}_j(x)=\dfrac{1}{n+1}\sum _{i=1}^n\mathbb {1}_{\{X_j^i\le x\}}$. The expressions of the densities ${\hat{f}}_{jj'}$ can be found in Sect. F.2 of the Supplementary Material. This approach does not require any parametric assumption on the marginals, and handles the cost of dimensionality by a pairwise approach. It also has the advantage of estimating ${\hat{\Sigma }}$ from the observed data in a single step.

In order to obtain the partial correlations, once an estimator ${\hat{\Sigma }}$ of *Σ * is obtained, it can be inverted via a penalized approach such as graphical Lasso (gLasso) [1] as shown in Eq. (4):

4 $$\begin{aligned} {\hat{\Omega }}_{\lambda }=\underset{\Omega }{\text {argmin}}\left( \text {tr}({\hat{\Sigma }}_{PD}\Omega ) - \log (|\Omega |) + \lambda ||\Omega ||_1\right) \end{aligned}$$

where ${\hat{\Sigma }}_{PD}$ denotes the projection of ${\hat{\Sigma }}$ on the space of positive definite matrices. In practice, this projection can be done via the algorithm provided in [15], which is implemented in the nearPD function from the Matrix R package. Note that the expression from Eq. (4) stems from maximum likelihood inference of *Ω * in the latent space as described in Sect. F.3 of the Supplementary Material. The optimal value of *λ * can be selected by minimizing the High-dimensional Bayesian Information Criterion (HBIC) [16] given in Eq. (5),

5 $$\begin{aligned} {HBIC(\lambda )=\text {tr}({\hat{\Sigma }}_{PD}{\hat{\Omega }}_{\lambda }) - \log (|{\hat{\Omega }}_\lambda |) + \log (\log (n))\dfrac{\log (d)}{n}K_\lambda }, \end{aligned}$$

where $K_\lambda$ denotes the number of non-null coefficients of ${\hat{\Omega }}_{\lambda }$.

### Comparison with a moment-based method

Another possible approach to estimate *Σ * is the use of bridge functions [10, 17, 18], which are based on a one-to-one correspondence between a statistic of the observed data, which we denote by *r*, and the matrix *Σ *. Note that this method does not require either any parametric assumption on the marginal distributions.

The first step consists in the estimation of *r*, which is a vector with as many components $r_{jj'}$ as there are pairs of variables $(X_j,X_{j'})$. For each pair, the estimator ${\hat{r}}_{jj'}$ of $r_{jj'}$ depends on the nature of the variables. When both variables $X_j$ and $X_{j'}$ are continuous, it actually coincides with Kendall’s *τ * and is computed as

6 $$\begin{aligned} {\hat{r}}_{jj'}=\dfrac{2}{n(n-1)}\sum _{1\le i < i' \le n}\text {sign}(X_j^i-X_j^{i'})\text {sign}(X_{j'}^i-X_{j'}^{i'}) .\end{aligned}$$

When $X_j$ is continuous and $X_{j'}$ is discrete, it is computed as

7 $$\begin{aligned} {\hat{r}}_{jj'}=\dfrac{2}{n(n-1)}\sum _{1\le i < i' \le n}\text {sign}(X_j^i-X_j^{i'})(X_{j'}^i-X_{j'}^{i'}). \end{aligned}$$

Finally, when the pair is discrete, it is computed as

8 $$\begin{aligned} {\hat{r}}_{jj'}=\dfrac{2}{n(n-1)}\sum _{1\le i < i' \le n}(X_j^i-X_j^{i'})(X_{j'}^i-X_{j'}^{i'}). \end{aligned}$$

In a second step, estimation of *Σ * is performed by solving ${B}({\hat{\Sigma }}_{jj'}) = {\hat{r}}_{jj'}$ for ${\hat{\Sigma }}_{jj'}$, where *B* denotes the so-called bridge function that makes the one-to-one correspondence. The expressions of the bridge functions for a continuous, discrete, mixed pair, denoted by ${B}^{cc}$, ${B}^{dd}$, ${B}^{dc}$, respectively, can be found in Sect. F1 of the Supplementary Material. See also [36].

However, several obstacles can arise. Indeed, the equation ${B}({\hat{\Sigma }}_{jj'}) = {\hat{r}}_{jj'}$ may not have any solution in the interval *[-1,1]*, in which case a manual correction may be needed. In this case, the solution is set to 1 (resp. *-1*) if positive (resp. negative). Moreover, the computation time of the bridge function increases with the number of categories of the discrete variables.

## Simulation study

The goal of this simulation study is to compare the performance of our proposed pairwise pseudo likelihood-based method and the moment-based approach with bridge functions.

### Simulation protocol

First, we simulate a partial correlation structure ${\tilde{\Omega }}$. We set a *d× d* matrix of zeroes and we choose a number *r* of non-null coefficients. Then, we randomly position *r*/2 non-null coefficients via a Watts-Storgatz approach [19] in the upper triangular part of the matrix. We fill them with uniform values ${\mathcal {U}}(0.2,0.7)$ and we symmetrize our matrix before setting its diagonal to 1. Finally, we make it nonnegative definite by adding the absolute value of its smallest eigenvalue to its diagonal. We compute ${\tilde{\Sigma }}={\tilde{\Omega }}^{-1}$ and standardize it to be a correlation matrix *Σ * as required in model (1). The detailed algorithm is presented in Sect. E of the Supplementary Material. Once generated, the matrices *Σ * and $\Omega =\Sigma ^{-1}$ are kept fixed throughout the whole simulation study.

We simulated a Gaussian vector ($Z_1, \dots , Z_d) \sim {\mathcal {N}}(0, \Sigma )$ of correlation matrix *Σ *. Then, we considered two simulation protocols in order to obtain our data set ($X_1, \dots , X_d$). The first one is similar to the setting presented by [10]. We simulated a data set that contains one half of Gaussian variables and another half of discrete variables with at most three categories. To do so, we simulated *d*/2 uniform cutoff values $C_{j1}\sim {\mathcal {U}}(0.25,0.85)$ and *d*/2 uniform cutoff values $C_{j2}\sim {\mathcal {U}}(1.5,2)$. We set $X_j = Z_j$ for *j=1, … , d/2*, and then we set $X_j = \mathbb {1}(Z_j> C_{j1}) + \mathbb {1}(Z_j > C_{j2})$ for *j=d/2+1 … , d*.

We then consider a more realistic second simulation protocol to complexify the structure of the dataset, in which one third of the variables are Gaussian ${\mathcal {N}}(0,1000)$, one third are discrete following a Negative Binomial distribution *NB*(1000, 0.3) and one third are binary ${\mathcal {B}}(\frac{1}{2})$. Note that Fig. S2 of the Supplementary Material shows the range of values taken by a Negative Binomial distribution for these parameters. For *j = 1, … , d*, we set $X_j = F_j^{\leftarrow }(\Phi (Z_j))$, where $F_j^{\leftarrow }$ corresponds to the generalized inverse of the corresponding marginal distribution.

In our simulation study, we have computed *N=100* replications, for $d\in \{30, 300\}$ variables, for sample sizes $n\in \{20, 50, 200, 500\}$. On each generated dataset, *Σ * was estimated according to the two inference methods from Sect. Methods. Then, a family of estimates ${\hat{\Omega }}_{\lambda }$ was calculated as in Eq. (4) for a grid of $\lambda \in \log \{1.01, 1.02 \dots , 2.99, 3\}$ for *d=30*, and $\lambda \in \{0.05, 0.06 \dots , 0.74, 0.75\}$ for *d=300*.

### Performance metrics

Three different performance metrics were computed in order to compare the two methods. First, the computational efficiency was evaluated by recording the CPU time for the computation of ${\hat{\Sigma }}$ and its inversion for one value of *λ *. Note that for the bridge functions estimation method (subsection Comparison with a moment-based method), the original code can be found on https://github.com/Aiying0512/LGCM and corresponds to the article by Zhang et al. [10]. We have parallelized it in order to have a fair comparison of computation time with the code for the PPMLE estimation method that is implemented in the heterocop R package [20], with 19 cores used by default.

Our main goal is to construct a biological network, in which an edge between two nodes corresponds to a non-null latent conditional correlation between the corresponding variables. A key step therefore consists in recovering the set of non-null conditional correlations between the latent variables. The simulated precision matrix *Ω * has a sparsity (proportion of zeroes) of about 0.8 for *d=30*, corresponding to 75 non-zeroes to be identified, and around 0.98 for *d=300*, corresponding to 750 non-zeroes. A zero corresponds to no conditional correlation, while a non-zero corresponds to a non-null conditional correlation. For each estimation ${\hat{\Omega }}_{\lambda }$, the penalization enables to set the estimated coefficients exactly to zero without a thresholding step. Note that the proportion of estimated zeroes increases along with the penalization parameter *λ *. For each *λ *, two main indicators were measured. The sensitivity, also known as true positive rate TPR*(λ )*, represents the proportion of correctly detected non-zeroes in ${\hat{\Omega }}_{\lambda }$ among the true non-zeroes in *Ω *. Similarly, the specificity, also known as the true negative rate TNR*(λ )*, corresponds to the proportion of correctly detected zeroes among the true zeroes. More specifically, we are going to consider the false positive rate, $FPR(\lambda )=1-TNR(\lambda )$. The Receiver Operating Characteristic (ROC) curve is a graphical representation of the *FPR* against the *TPR* depending on the parameter *λ *. When *λ * is high, ${\hat{\Omega }}_{\lambda }$ is strongly penalized and all non-diagonal coefficients are zero, leading to *TPR(λ )=0* and *FPR(λ )=0*. When *λ * is low, ${\hat{\Omega }}_{\lambda }$ is not penalized and there might be no zero coefficients, leading to *TPR(λ )=1* and *FPR(λ )=1*. The Area Under Curve (AUC) criterion computes the area under the ROC curve in order to evaluate the performance of the classifier.

Finally, to assess the performance of the estimator ${\hat{\Sigma }}$, we shall examine the distribution of the squared distances between the replicated estimates ${\hat{\Sigma }}^k$, *k=1, … , N* and the true *Σ *, given by:

9 $$\begin{aligned} \sum _{j < j'} (\Sigma _{jj'} - {\hat{\Sigma }}^k_{jj'})^2. \end{aligned}$$

### Simulation results

First, the results are presented for *d=30* variables, for both simulation protocols, for *N=100* replications. It has to be noted that additional results, in terms of computing time and estimation accuracy are provided in Figs. S3 and S4 of the Supplementary Material for various values of the negative binomial parameter for the bridge function approach, which was found to be highly sensitive to these values.

#### Comparison of computation times

**Fig. 1:**
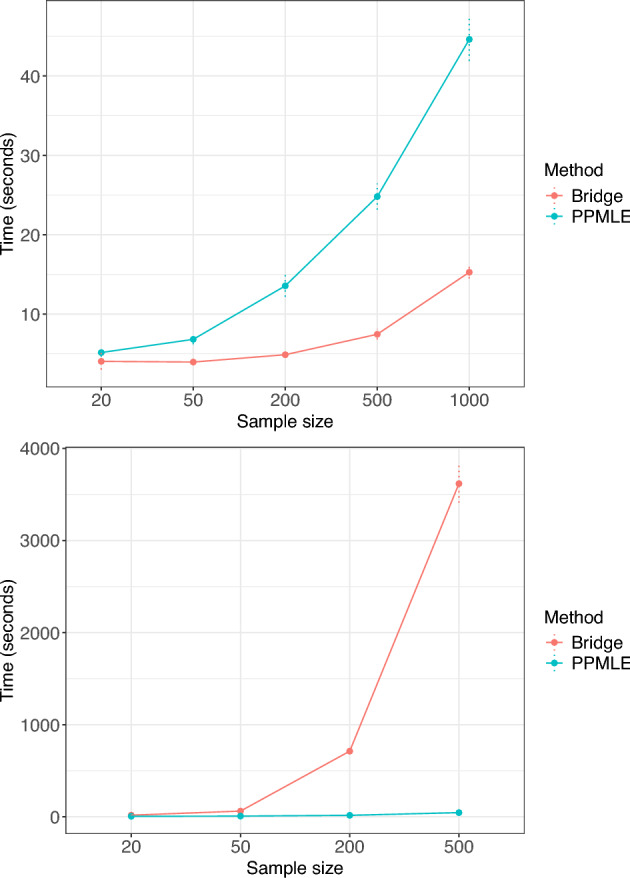
Computation time for *d=30* variables, for both methods, for the first simulation protocol (top) and for the second simulation protocol (bottom)

Figure 1 shows that for the first simulation protocol, when the discrete variable only has three categories $\{0, 1, 2\}$, the bridge function performs similarly until *n=200*, and slightly better for larger sample sizes *n*, needing under 10 s per iteration when *n=500* while the PPMLE estimation needs about 30 s for this sample size. A much larger difference is observed in the second simulation protocol. Indeed, this simulation setting leads to a discrete variable that can take several dozens of discrete values between 2000 and 2500. In this case, the computational time of the bridge function method drastically increases from around two minutes for *n=50* to over ten minutes when *n=200*, and close to an hour per iteration for *n=500*, while the PPMLE estimation remains, on average, under two minutes for all sample sizes. Note that the complexity of the PPMLE algorithm is linear in *n* and quadratic in *d*.

#### AUC values

The presence of conditional correlations has been detected via ROC curves, which AUC values for both simulation protocols and for both methods are given in Table 1. We can see that in the case of the first simulation protocol, the results are quite similar, even if the PPMLE estimation seems to perform slightly better for *n=20* and *n=50*. Larger differences are observed for the second scenario, especially when discrete variables are involved, as presented in Table 2. An explanation might be that bridge functions are not well suited for the analysis of categorical data when the number and values of the categories can be large, which is typically the case for count data, especially for RNA-seq data which are often assumed to come from negative binomial distributions. As expected, AUC values increase with the sample sizes. As shown in Table 2, PPMLE performances, however, still remain robust even for small sample sizes (n = 20), particularly for continuous–discrete variables.

**Table 1:** Estimated *AUC* for *d=30* variables computed from *N=100* replications, for each method, sample size and scenario

	Sample size	20	50	200	500
Scenario 1	PPMLE	0.66 (0.03)	0.70 (0.02)	0.76 (0.01)	0.78 (0.01)
	Bridge	0.64 (0.02)	0.67 (0.02)	0.75 (0.01)	0.77 (0.02)
Scenario 2	PPMLE	0.68 (0.008)	0.72 (0.01)	0.77 (0.007)	0.78 (0.006)
	Bridge	0.64 (0.01)	0.66 (0.01)	0.72 (0.007)	0.76 (0.004)

The standard errors of each estimate are given in parentheses

**Table 2:** Estimated *AUC* for *d=30* variables computed from *N=100* replications, for each method, sample size and scenario, for the discrete-discrete and continuous-discrete (CD) pairs of variables

	AUC on discrete pairs		AUC on CD pairs	
Sample size	PPMLE	Bridge	PPMLE	Bridge
20	0.64 (0.03)	0.60 (0.03)	0.84 (0.03)	0.73 (0.04)
50	0.70 (0.02)	0.60 (0.02)	0.91 (0.01)	0.76 (0.02)
200	0.76 (0.02)	0.64 (0.02)	0.93 (0.004)	0.83 (0.02)
500	0.78 (0.01)	0.67 (0.007)	0.93 (0.001)	0.92 (0.001)

The standard errors of each estimate are given in parentheses

#### Squared error

**Fig. 2:**
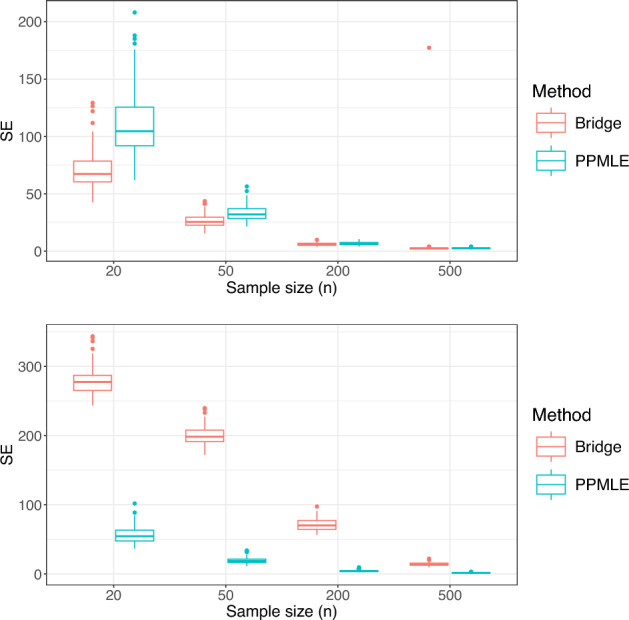
Boxplot of the *N=100* SE values for *d=30* for scenarios 1 (top) and 2 (bottom)

**Fig. 3:**
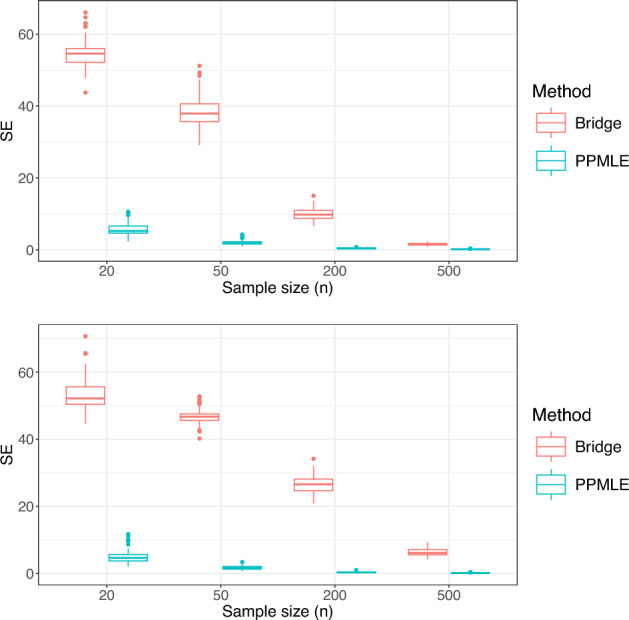
Boxplot of the *N=100* SE values for *d=30* for continuous-discrete (top) and discrete-discrete (bottom) pairs in the second simulation scenario

The overall Squared Error (SE) values, computed as in Eq. (9) for both simulation scenarios, are given in Fig. 2. In the first simulation scenario, both methods perform similarly, except for a sample size of 20 for which the bridge functions are slightly better. On the other hand, for the second simulation scenario, for which the discrete data have larger values and number of categories, much better performances are observed for the proposed PPMLE approach, as shown in Fig. 2. This difference is even more drastic in the presence of discrete variables, either for discrete-discrete pairs or continuous-discrete pairs, as presented in Fig. 3. For binary variables, the SE values are quite similar for both methods, as shown in Fig. S1 of the Supplementary Material.

#### Simulation results in high dimension

**Fig. 4:**
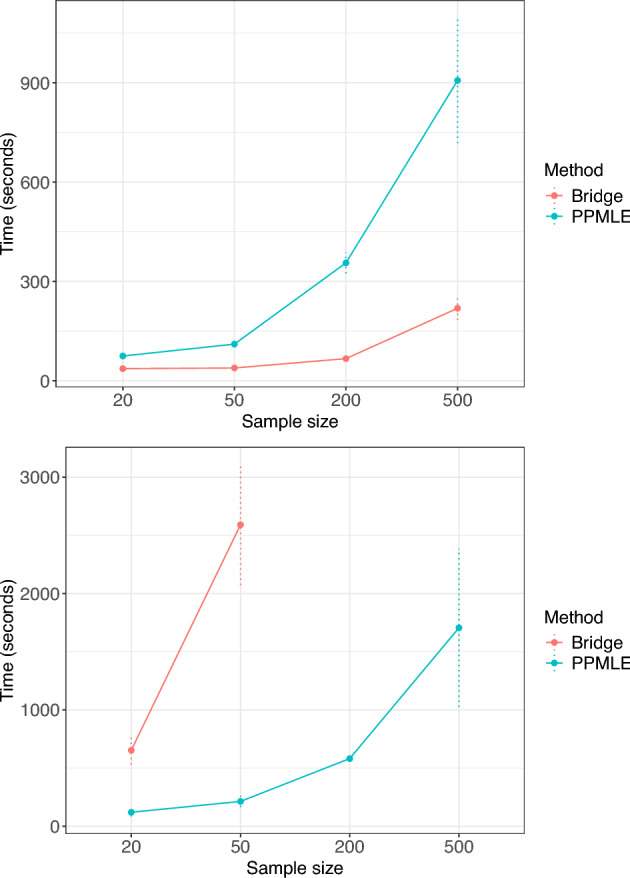
Computation time for *d=300* variables, for both methods, for the first simulation protocol (top) and for the second simulation protocol (bottom). Sample sizes *n=200, 500* have not been considered for the Bridge functions estimator because of the high computational time.

Simulations were run for a larger number of variables (*d=300*). Fig. 4 shows the average computation time for both simulation scenarios and both estimation methods. For the first simulation scenario where the discrete variables only take values in $\{0,1,2\}$, the computation time is lower for the bridge function estimator for all sample sizes. Both remain under five minutes for *n=20,50,200*. In the second simulation scenario, on the other hand, in which the discrete variables have a much larger number of categories, the computational time required for the bridge function approach is much larger than for the PPMLE approach, which prevents to run this method for sample sizes larger than 50 in this simulation study.

**Fig. 5:**
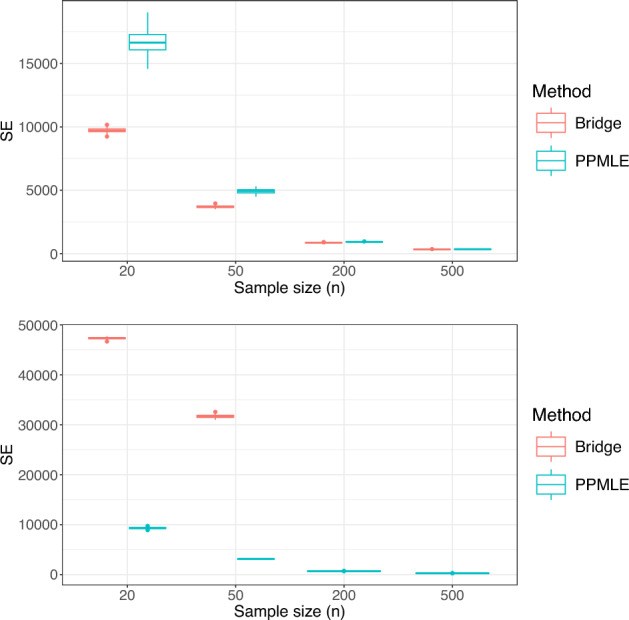
Boxplot of the *N=100* SE values for *d=300* for scenarios 1 (top) and 2 (bottom)

The estimated AUC values and the distributions of the SE values are presented in Table 3 and Fig. 5. Both methods present similar results for the first simulation scenario. For the second scenario, the bridge function approach could not be evaluated for sample sizes larger than *n=50*, due to the required computing time, which limits the interpretation of the results. Our approach performs well in terms of AUC values and SE even for *d=300* variables for the different sample sizes evaluated, up to *n=500*. The computational time required for the bridge functions for the analysis of such data is, however, a great difficulty as it prohibits its use for real data analysis, such as RNA-seq data, as presented in the section below.

**Table 3:** Estimated AUC for *d=300* variables computed from *N=100* replications, for each method, sample size and scenario

	Sample size	20	50	200	500
Scenario 1	PPMLE	0.63 (0.006)	0.71 (0.004)	0.84 (0.004)	0.90 (0.003)
	Bridge	0.62 (0.005)	0.70 (0.005)	0.84 (0.004)	0.90 (0.003)
Scenario 2	PPMLE	0.65 (0.004)	0.74 (0.004)	0.87 (0.004)	0.92 (0.002)
	Bridge	0.64 (0.003)	0.70 (0.004)	–	–

The standard errors of each estimate are given in parentheses

Additional simulation results are presented in the Supplementary Material Tables S1 and S2 with negative partial correlation values. Similar conclusions were obtained regarding the comparison between PPMLE and the bridge functions. A simple simulation setting was added to investigate the behavior of the classical glasso and highlight its difficulties to deal with non-Gaussian data, in particular in the case of rare events binary data (see Figs. S11 and S12 as well as Tables S3 and S4 of the Supplementary Material).

## Real data application

To evaluate the practical performance of our proposed inference method, we applied it to breast cancer multi-omics data from the International Cancer Genome Consortium (ICGC) [11]. This dataset contains various molecular profiles, including gene expression, protein abundance and mutation data, providing a comprehensive view of tumor heterogeneity. Note that proteomics data was obtained via a targeted assay from MD Anderson Reverse Phase Protein Array. Our objective was to infer regulatory interactions between these molecular features and identify key network structures associated with breast cancer. The pre-processed dataset used in this study is available on github [21]. Our variables of interest includes protein expression (continuous), RNA sequencing (discrete) and mutations (binary) measured on breast cancer tumoral tissue. The initial data set included normalized protein abundance for 115 genes measured on 260 individuals, RNA-seq counts for 20 501 genes observed on 939 individuals, and presence of 107 249 mutations observed on 918 individuals. As there were several samples per individual, we averaged the RNA-seq and protein expression values. The binary variables encoded if the mutations were present for at least one of the samples. Then, we used the DESeq2 R package [22] to normalize the RNA-seq counts. The intersection of available data for all type of variables left us with 250 individuals. Finally, the 108 genes found in common between the RNA-seq and protein data were kept, as well as the 62 mutations that were present in at least two donors. Note that among the 62 mutations, 58 are present on genes that are not concerned by the RNA-seq and protein expression measurements. In the end, the final dataset contained 278 variables (108 protein expression, 108 RNA-seq counts, 62 mutations) observed on 250 individuals. Note that our RNA-seq counts have a median value of 3706.

The precision matrix *Ω * was inferred via the graphical lasso applied to the estimate of the copula correlation matrix obtained from pseudo pairwise likelihood estimation. Estimates ${\hat{\Omega }}_{\lambda }$ of *Ω * were computed for each value of *λ * in a grid with values between zero and one. As the dataset comprise count data with a large number of distinct values, and large values of counts, the method of bridge functions was too computationally expensive and could not be applied.

Figure S5 from the Supplementary Material shows the proportion of kept edges per variable type depending on the values of the penalization parameter *λ *. We can see that the proportion of detected edges decreases faster for RNA-protein and mutation-mutation pairs, while the other curves behave similarly.

**Fig. 6:**
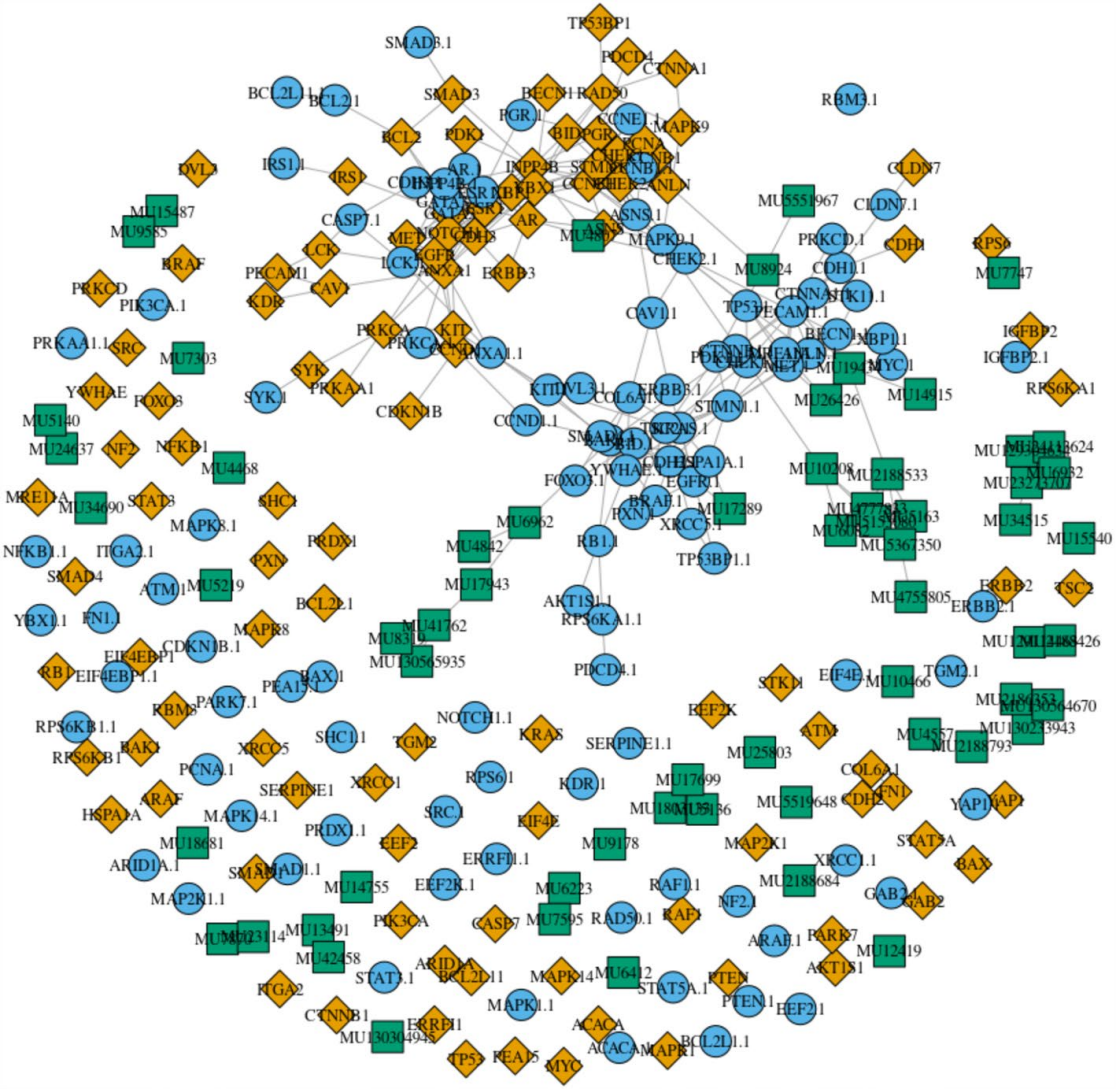
Estimated network between the RNA-seq counts (yellow diamonds), proteins (blue circles) and mutations (green squares) with the optimal value of *λ =0.53* returned by the HBIC criterion

The HBIC criterion was used to choose the penalization parameter, and an optimal value of *λ =0.53* was found, as illustrated in Fig. S6 from the Supplementary Material. The conditional correlation graph obtained for this optimal value is given in Fig. 6. The package igraph [35] was used to plot the networks.

Table 4 shows the number of edges per variable type. As shown in Fig. 6 and Table 4, the larger number of links identified in the network is within proteins for one major cluster, and within RNA-seq counts for a second major cluster. Note that both clusters are strongly connected, with 64 links between them. A fewer number of links are identified within mutations (45), and only 9 (resp. 4) links between mutations and proteins (resp. genes).

**Table 4:** Number of edges for the optimal lambda

Variable type	Number of edges
Protein-protein	125
RNA-RNA	106
RNA-protein	64
Mutation-mutation	45
Protein-mutation	9
RNA-mutation	4
Total	353

**Fig. 7:**
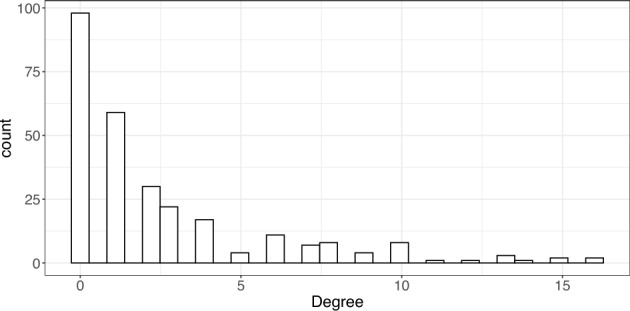
Histogram of the degrees of the nodes in the selected network

**Fig. 8:**
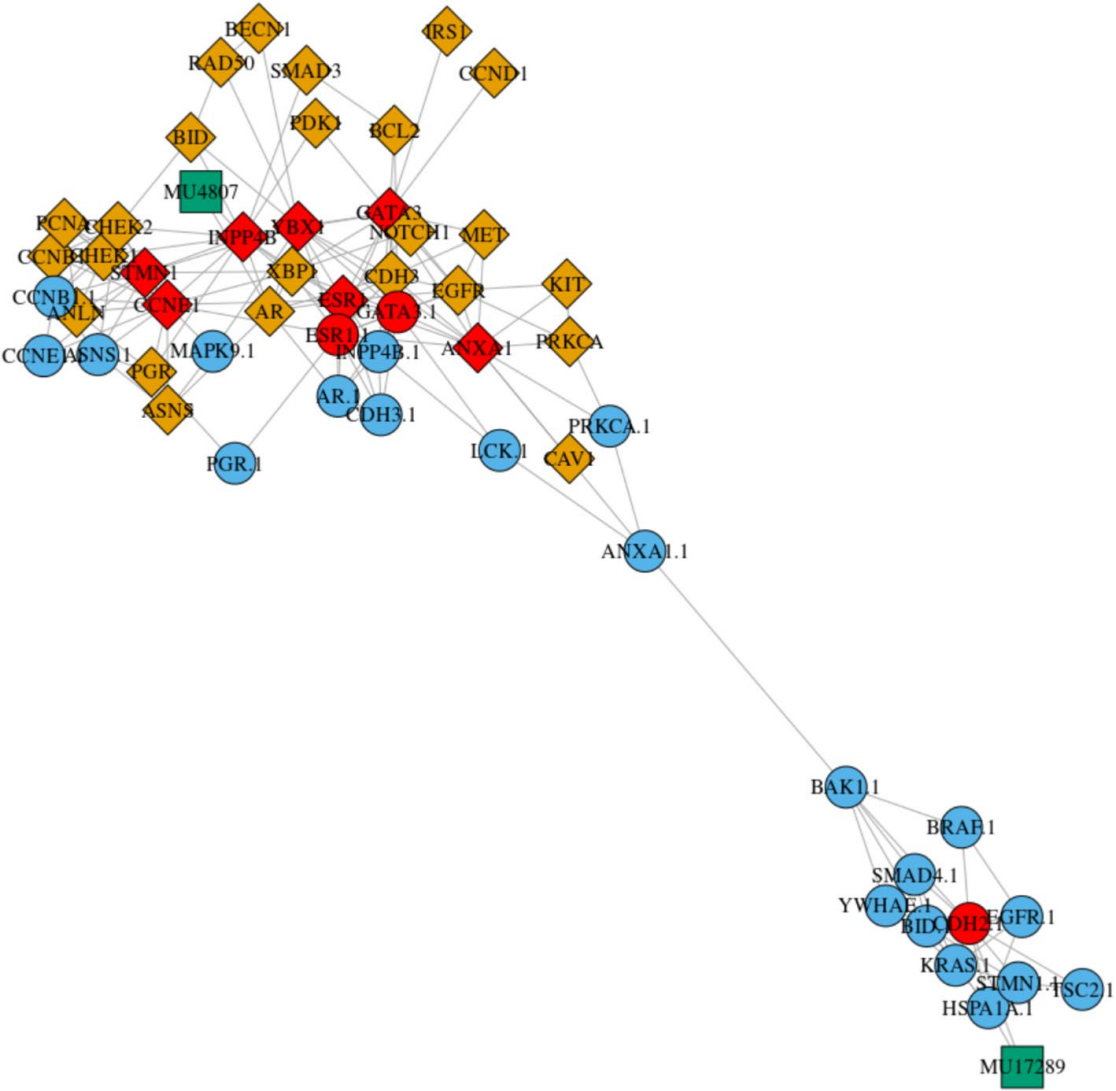
Subgraph corresponding to the neighborhoods of genes (resp. proteins) from Table 5, represented as red diamonds (resp. circles). The mutations are represented as green squares, the other genes as yellow diamonds and the other proteins as blue circles

Figure 7 shows a histogram of the degrees of the nodes in the obtained graph. Only ten of them have over ten connected edges. Moreover, Fig. S7 from the Supplementary Material provides a histogram of the degree distribution for each omics type (protein, RNA, mutations). Although the distributions for the RNA and proteins are quite similar, the mutations seem to have less connected egdes, with a maximum degree of six. These nodes correspond to the genes and proteins given in Table 5. It is interesting to note that all of them have been found to be related to breast cancer. The scientific references presenting these results are given in Table 5. Furthermore, for several of these nodes, we identified both the gene and the related protein. It is the case for GATA3 and ESR1. Figure 8 presents the subgraph corresponding to the neighborhoods of genes and proteins from Table 5. Overall, the nodes with the highest number of edges are linked to each other in two main hubs: one consisting of proteins, the other one of mostly RNA-seq counts. Two mutations are included in the graph: MU4807 and MU17289. They are related to genes TP53 and CDKN1B, which are known to have an influence on breast cancer [23, 24]. One of the scientific articles [25] also showed a link between gene CCNE1 and gene ANLN. This link was successfully identified with our network inference procedure as shown in Fig. 8. It is highlighted in Fig. S8 of the Supplementary Material and present in the corresponding adjacency matrix from Fig. S9.

**Table 5:** Genes and proteins with degree larger than ten

Variable	Type	Degree	References
CCNE1	RNA-seq	16	Marra [26]
GATA3	RNA-seq	16	Takaku [27]
ESR1	RNA-seq	16	Brett [28]
INPP4B	RNA-seq	15	Rodgers [29]
YBX1	RNA-seq	14	Shibata [30]
ANXA1	RNA-seq	13	Al Ali [31]
ESR1	Protein	13	Brett [28]
GATA3	Protein	13	Takaku [27]
STMN1	RNA-seq	12	Liu [32]
CDH2	Protein	11	Sinha [33]

**Fig. 9:**
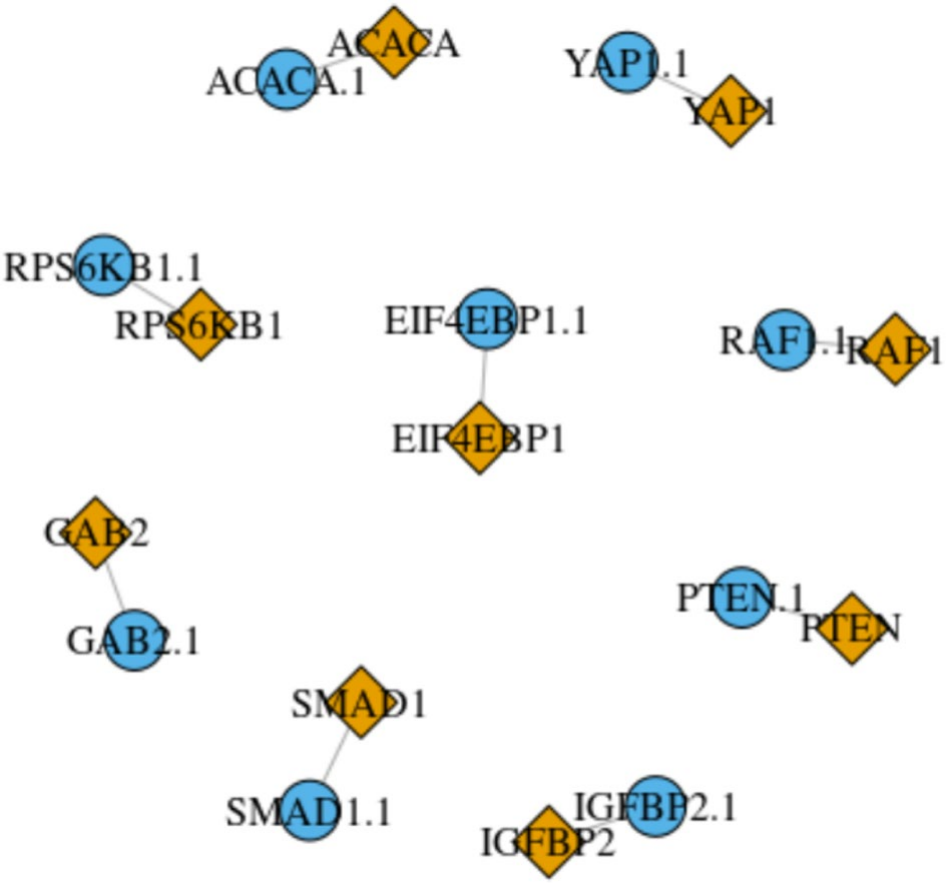
Pairs of proteins (blue circles) and RNA-seq (yellow diamonds) not linked to any other node in the main graph presented in Fig. 6

Additionally, among the nodes outside the main clusters of the network in Fig. 6, a few independent pairs of proteins and genes have been identified and are presented in Fig. 9. It is interesting to notice that each of these pairs of RNA-seq and protein corresponds to a single gene.

Additional results are presented in the Supplementary Material in Figs. S13 to S16, with an application of the bridge functions and the glasso approach to the real data. As shown in the previous section, the bridge functions were not able to deal with discrete RNA-seq data, with large values and a high number of categories. On the other hand, as in the simulation results, the glasso was not able to analyze properly the rare event binary data, and detected no links involving mutations.

## Discussion

In this study, we proposed a Gaussian copula model for multi-omics network inference, with a pairwise-likelihood estimation and graphical lasso regularization. In an extensive simulation study, we showed that the proposed pairwise-likelihood estimation method proved to be more effective as compared to moment-based methods using bridge functions. Its computational time is not impacted by the analysis of discrete variables with a large number of categories. It performs well in terms of AUC even in the case of large discrete values, which are poorly handled by the bridge functions estimation.

The above results have shown relevance of PPMLE estimation as opposed to bridge functions when inferring biological networks that involve, for instance, RNA-seq count data which often have large values and many categories. When applied to ICGC breast cancer data, our method has successfully identified biologically relevant interactions. For instance, it recovered direct links between proteins and RNA-seq counts belonging to a same gene. It also highlighted hubs of biologically important variables linked to breast cancer.

Several challenges will be worthwhile investigating in future work. The selection of the optimal regularization parameter remains crucial for balancing sparsity and accuracy in network estimation. We proposed in this study to use the HBIC selection criterion, but further exploration of adaptive penalization techniques may enhance model selection.

Overall, our approach provides a computationally efficient and robust tool for network inference in high-dimensional multi-omics data, enriching the toolkit for genomic data analysis.

## Supplementary Information

Supplementary Material 1.

## Data Availability

The dataset analysed during the current study is available in the data.csv file in the “Supplementary material for Multi-omics network inference with a Gaussian copula model” repository, https://github.com/ekaterinatomilina/network-copula.
