# Supplementary material for: Multi-omics network inference with a Gaussian copula model
Source: BMC Bioinformatics. 2026 Jun 4;27:174. doi: 10.1186/s12859-026-06502-3 (PMC13465878; doi:10.1186/s12859-026-06502-3)
Supplement: Supplementary file 1 [file 12859_2026_6502_MOESM1_ESM.pdf]

Supplementary Material

A Supplementary Figures

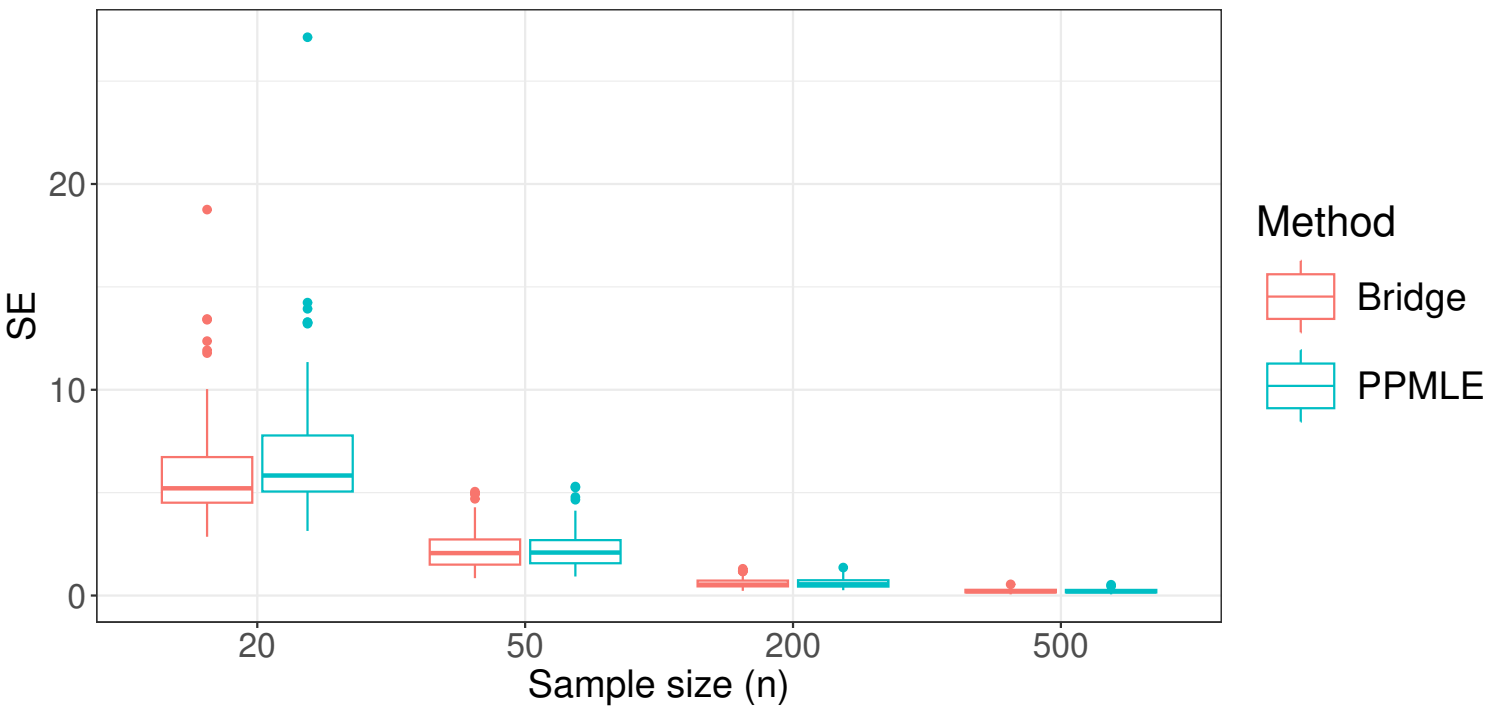

Fig. S1: Boxplots of the $N = 100$ SE values for $d = 30$ for binary-binary pairs in the second simulation scenario.

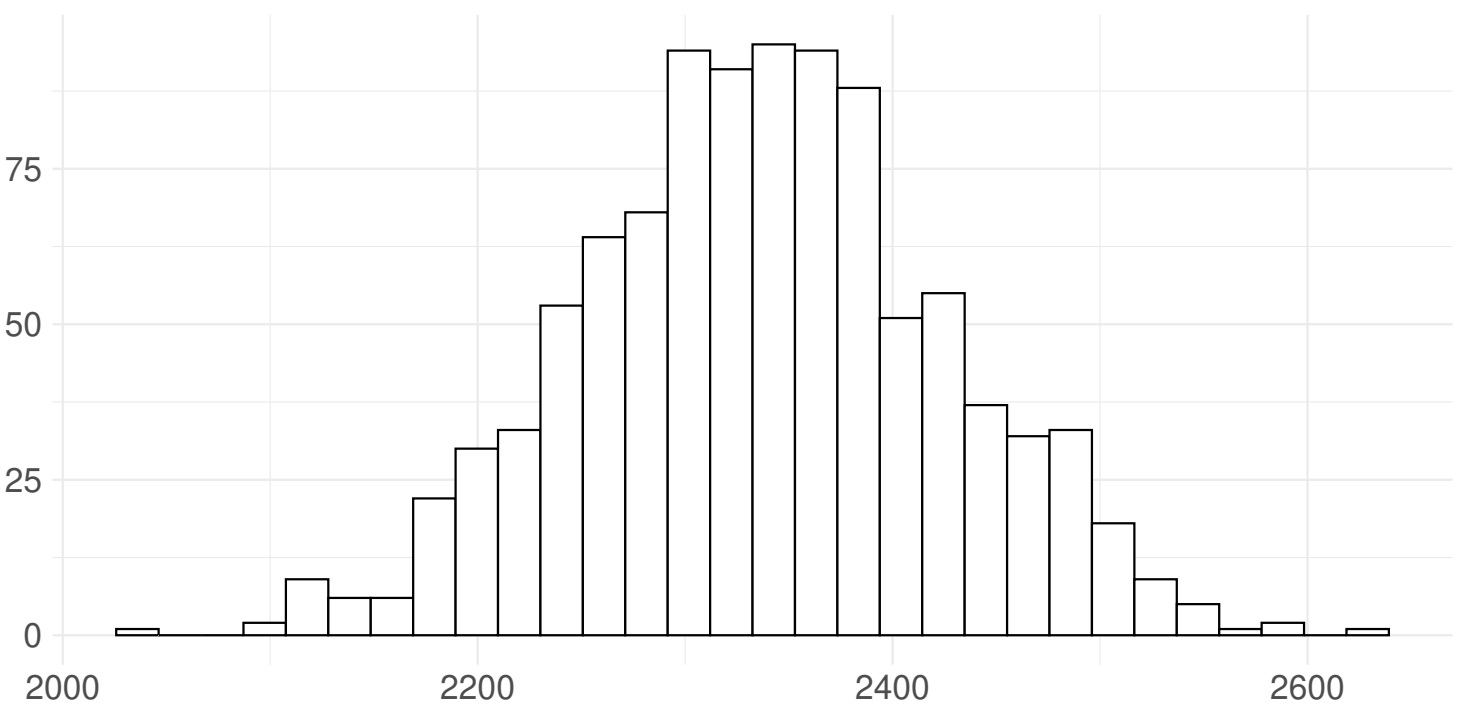

Fig. S2: Values taken by the negative binomial distribution of parameters $s = 1000$, $p = 0.3$ for a sample size $n = 1000$.

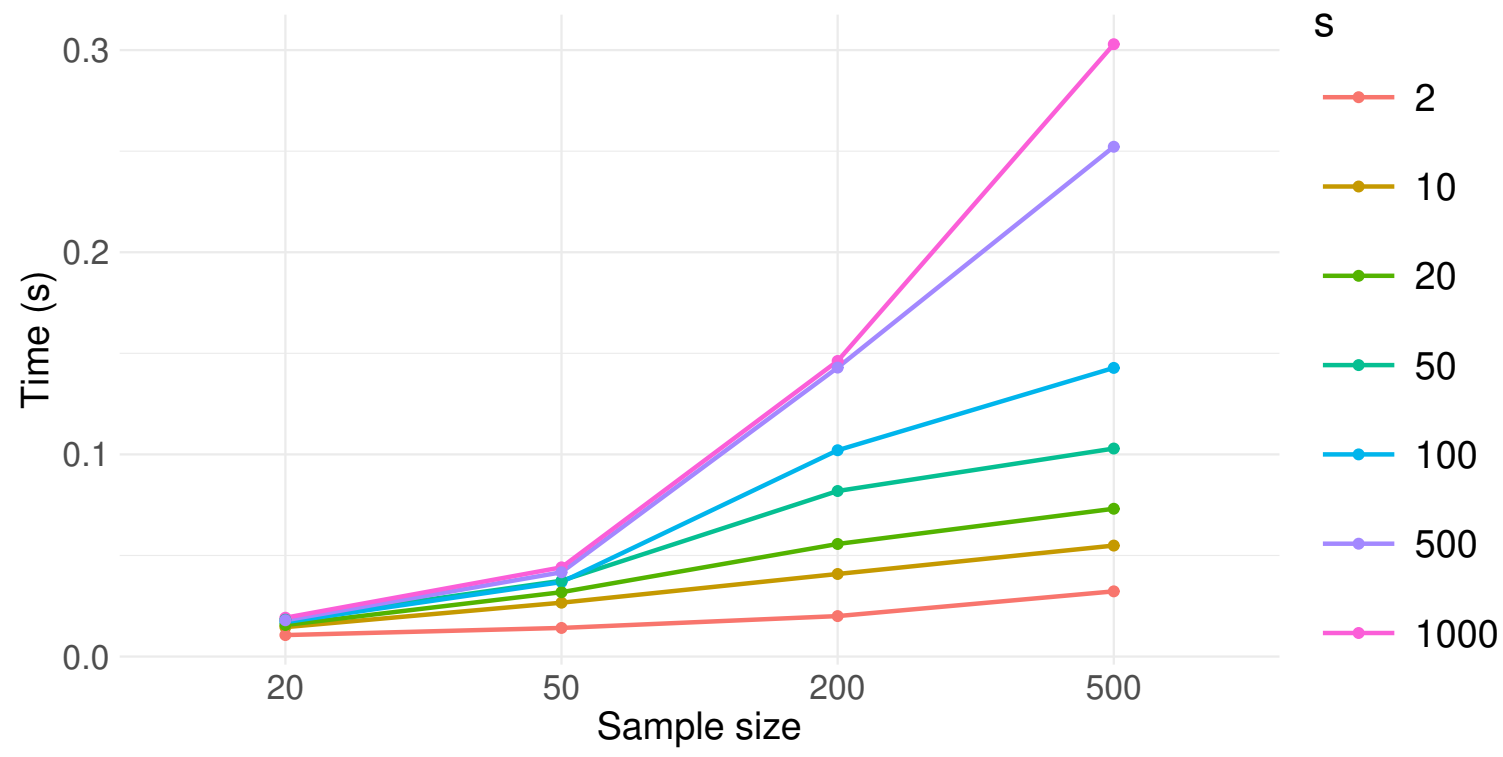

Fig. S3: Average computational time for the estimation of the copula correlation coefficient $\hat{\Sigma}_{12}$, $N = 100$ iterations by the bridge function estimator between a pair of variables of distributions $\mathcal{N}(0, 1000)$ and $NB(s, 0.3)$ with $s \in \{2, 10, 20, 50, 100, 500, 1000\}$, for various sample sizes.

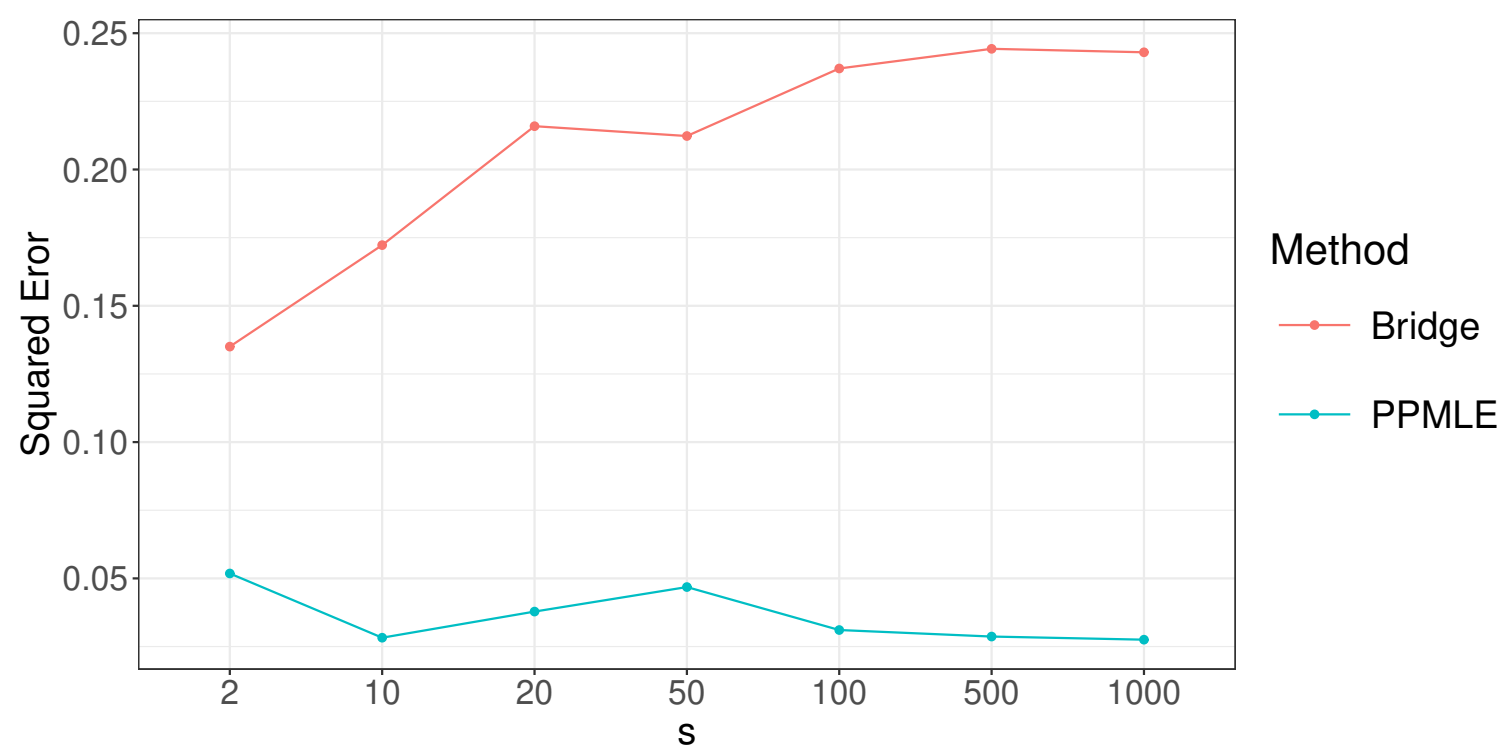

Fig. S4: Averaged squared error between the true copula correlation parameter $\Sigma_{12} = 0.5$ and the estimated parameter $\hat{\Sigma}_{12}$, for $N = 100$ iterations, by the bridge function estimator between a pair of variables of distributions $\mathcal{N}(0, 1000)$ and $NB(s, 0.3)$ with $s \in \{2, 10, 20, 50, 100, 500, 1000\}$, for a sample size of $n = 20$. Note that the number of categories in the Negative Binomial distribution increases with the value of parameter s. While the PPMLE always seems to perform well, the average squared error of the bridge function increases with the s value, and therefore with the number of categories of the discrete variable. It reaches a maximum of 0.25, which can be explained by the projection on -1, 1 when the bridge function does not have a root in $[-1, 1]$, leading to $\hat{\Sigma}_{12} = 1$ and so $(\hat{\Sigma}_{12} - \Sigma_{12})^2 = (1 - 0.5)^2 = 0.25$.

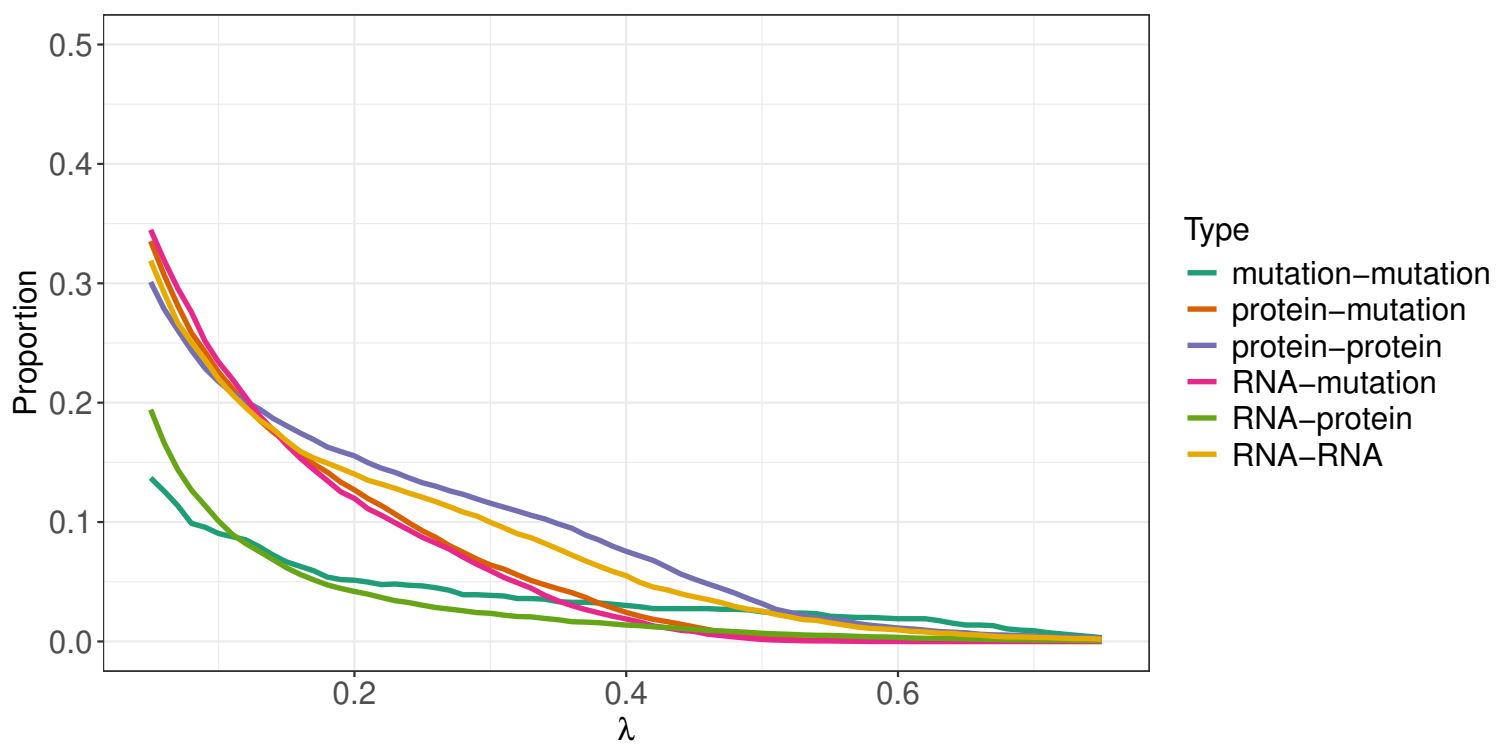

Fig. S5: Proportion of detected edges for each pair of variable types as a function of the penalization parameter λ.

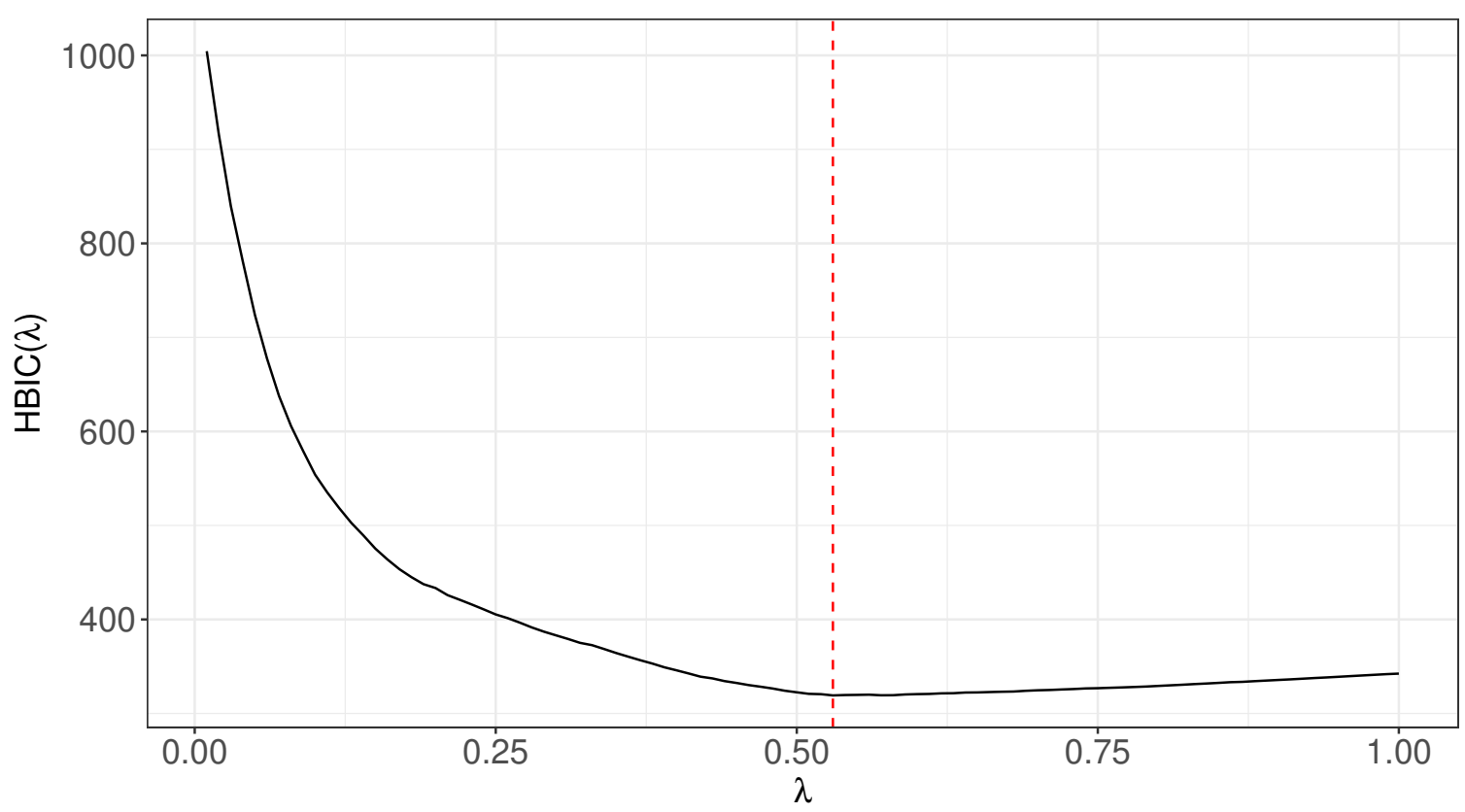

Fig. S6: Values of HBIC(λ), $0.05 < \lambda \leq 0.75$, for the real data analysis with the PPMLE method.

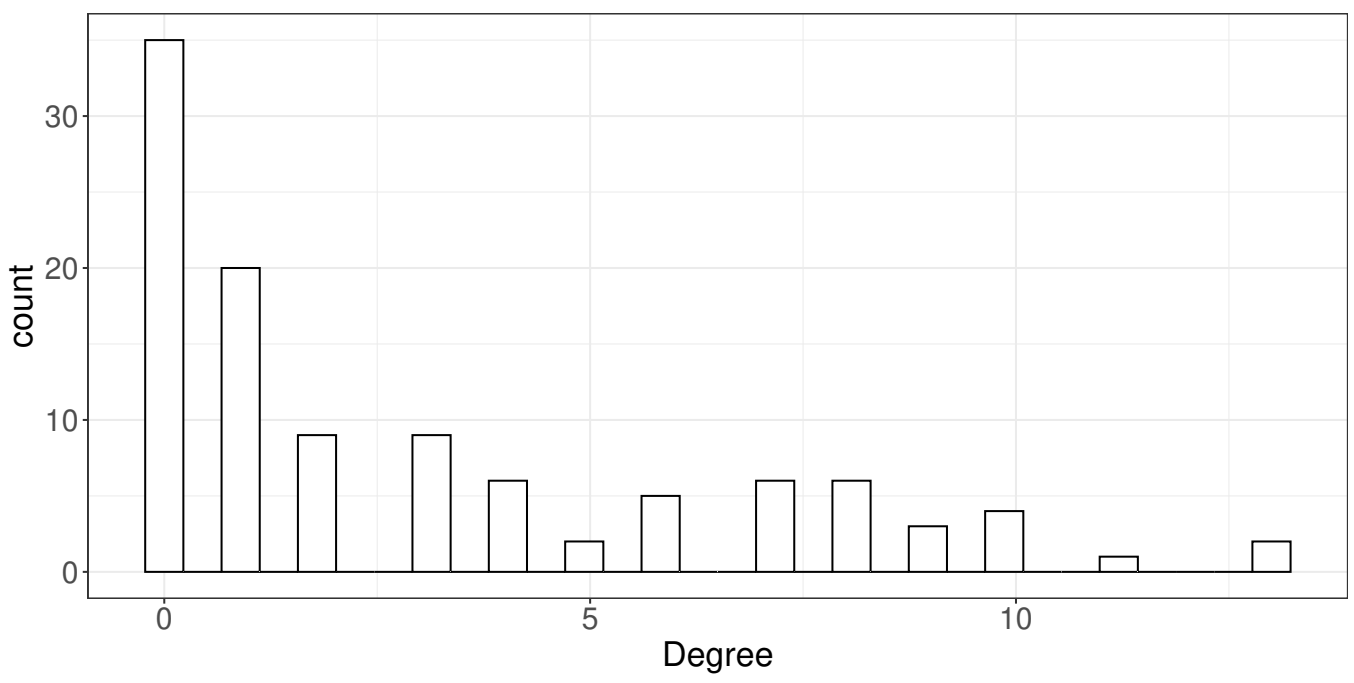

(a) RNAseq

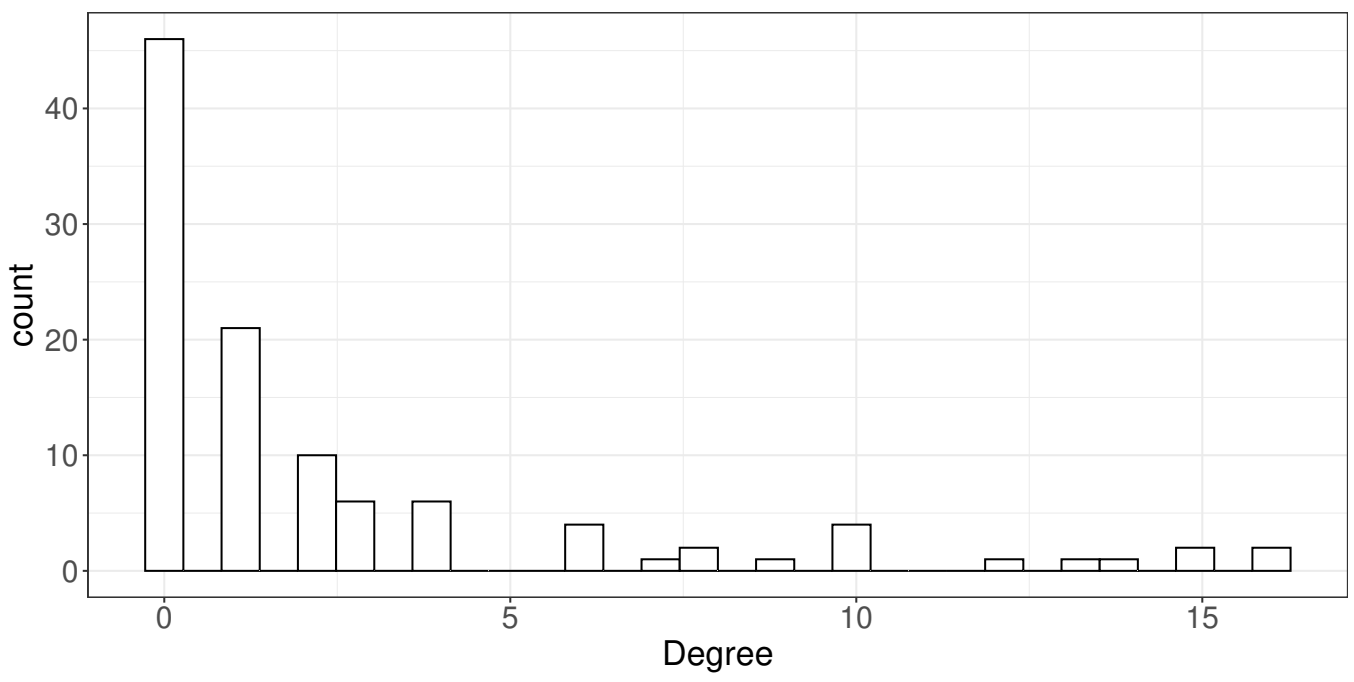

(b) Proteins

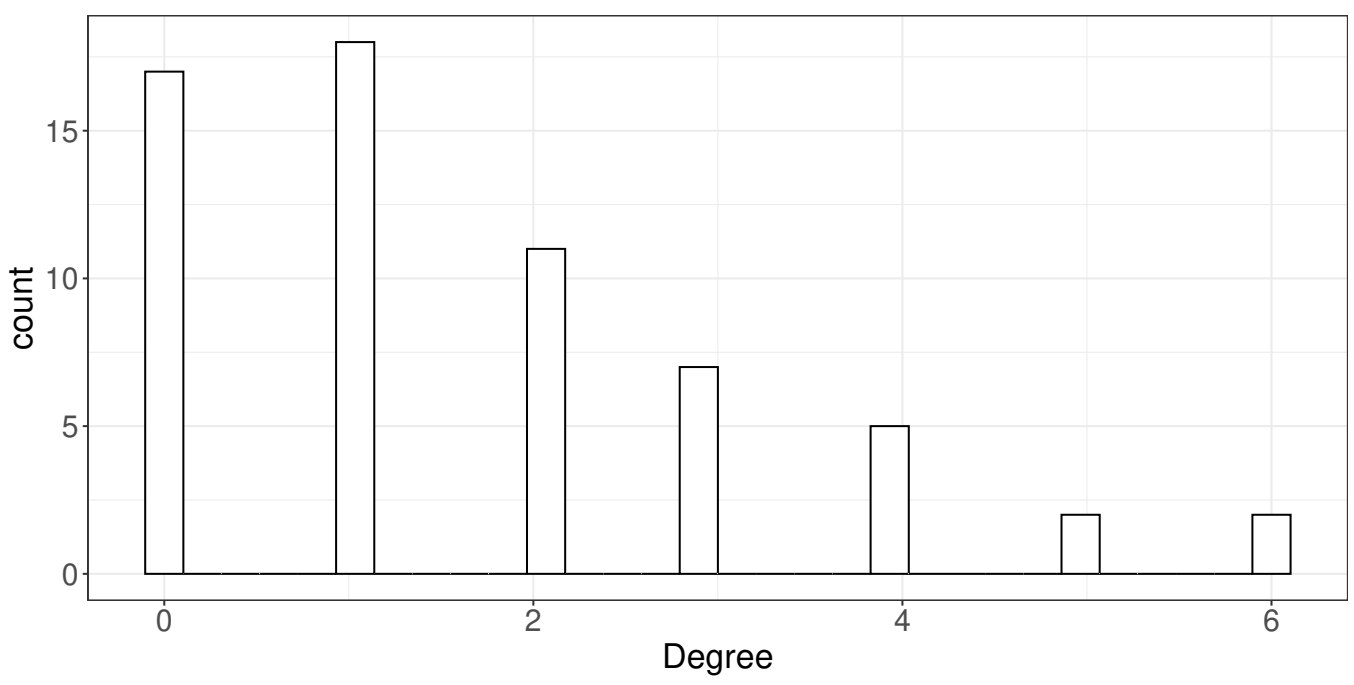

(c) Mutations

Fig. S7: Distribution of the degrees of the vertices from Figure 6 of the main text according to the omics type: RNAseq (top), protein (middle), mutations (bottom).

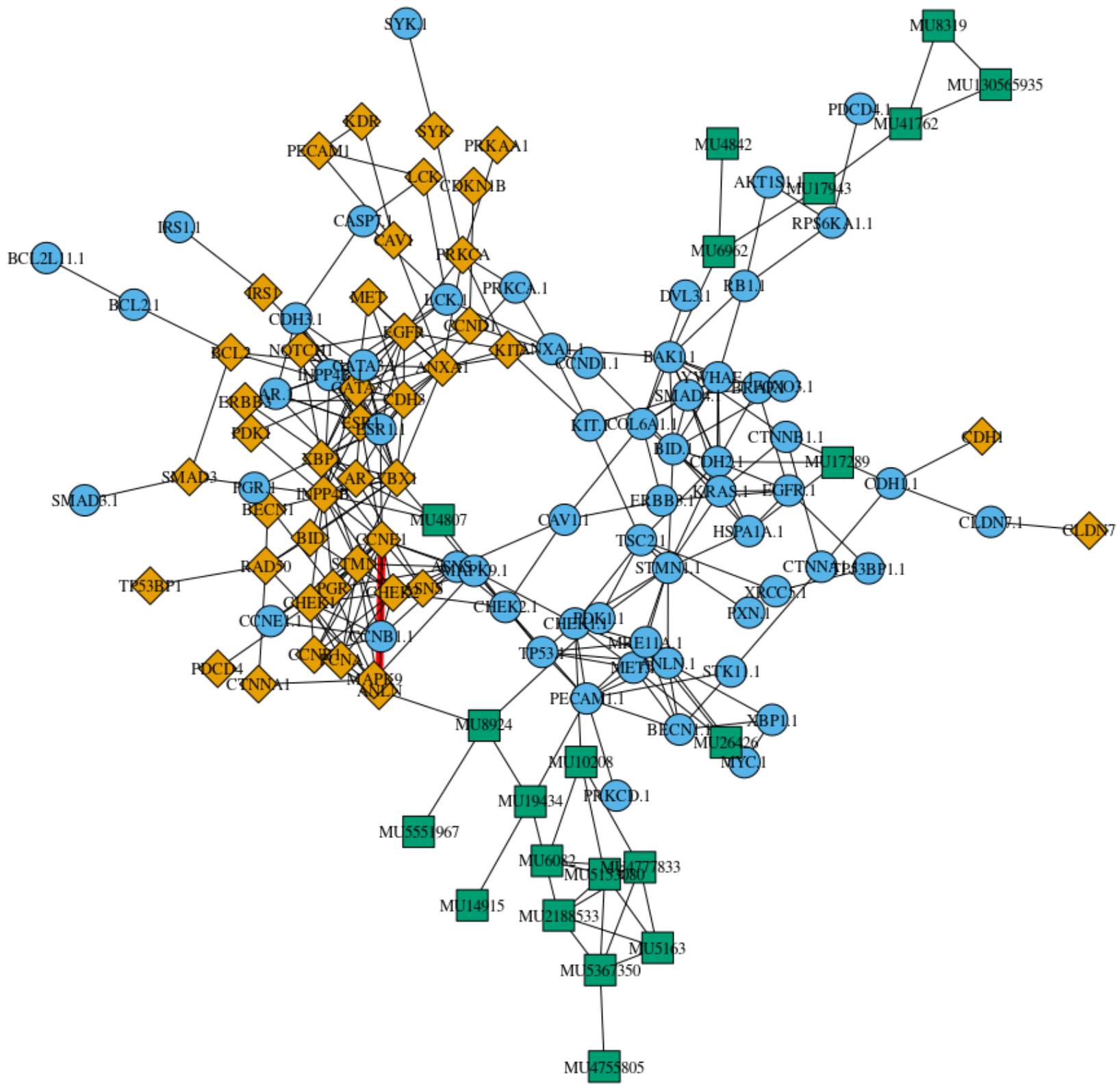

Fig. S8: Largest connected subgraph from Figure 6 of the main text. The highlighted bold red edge corresponds to the link between genes CCNE1 and ANLN, mentioned in [26].

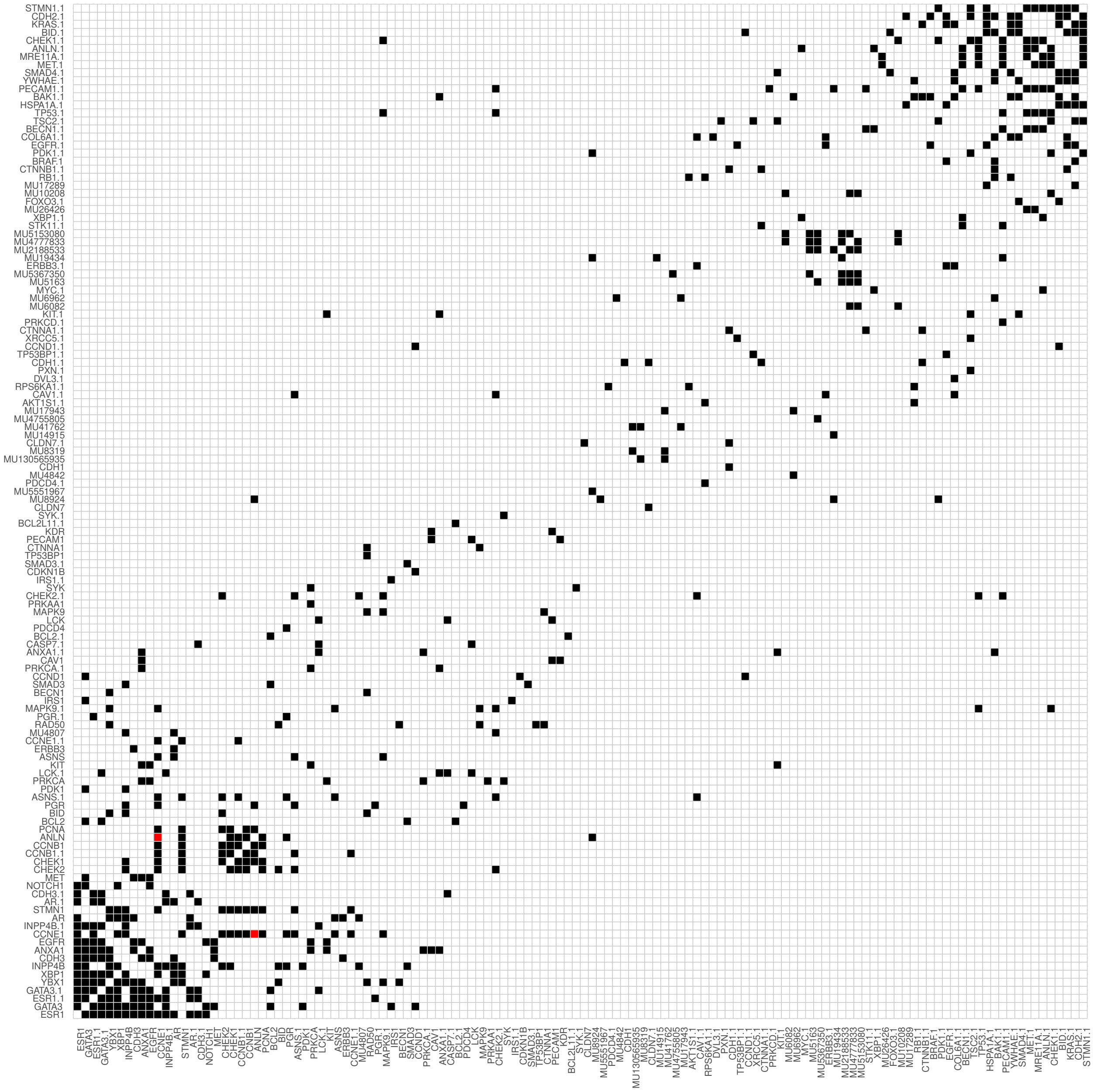

Fig. S9: Adjacency matrix corresponding to Figure S8. The colored red square corresponds to the edge between ANLN and CCNE1.

B Additional simulations

B.1 Comparison with the bridge functions for negative values of Ω

We have compared our estimation method (PPMLE) to the bridge functions for negative partial correlation values. This simulation was performed $N = 500$ times for $d = 30$ variables which marginal distributions corresponded to the second simulation protocol in Section 3. This time, they were linked by a Gaussian copula model of precision matrix Ω that also contained negative values, by considering a $\mathcal{U}[-0.7, 0.7]$ distribution in the algorithm given in Section E of the Supplementary Material. As shown in Tables S1 and S2, the conclusions obtained regarding the comparison between PPMLE and the bridge functions remain similar to the second simulation protocol in Section 3 and are robust to the presence of negative values in the precision matrix.

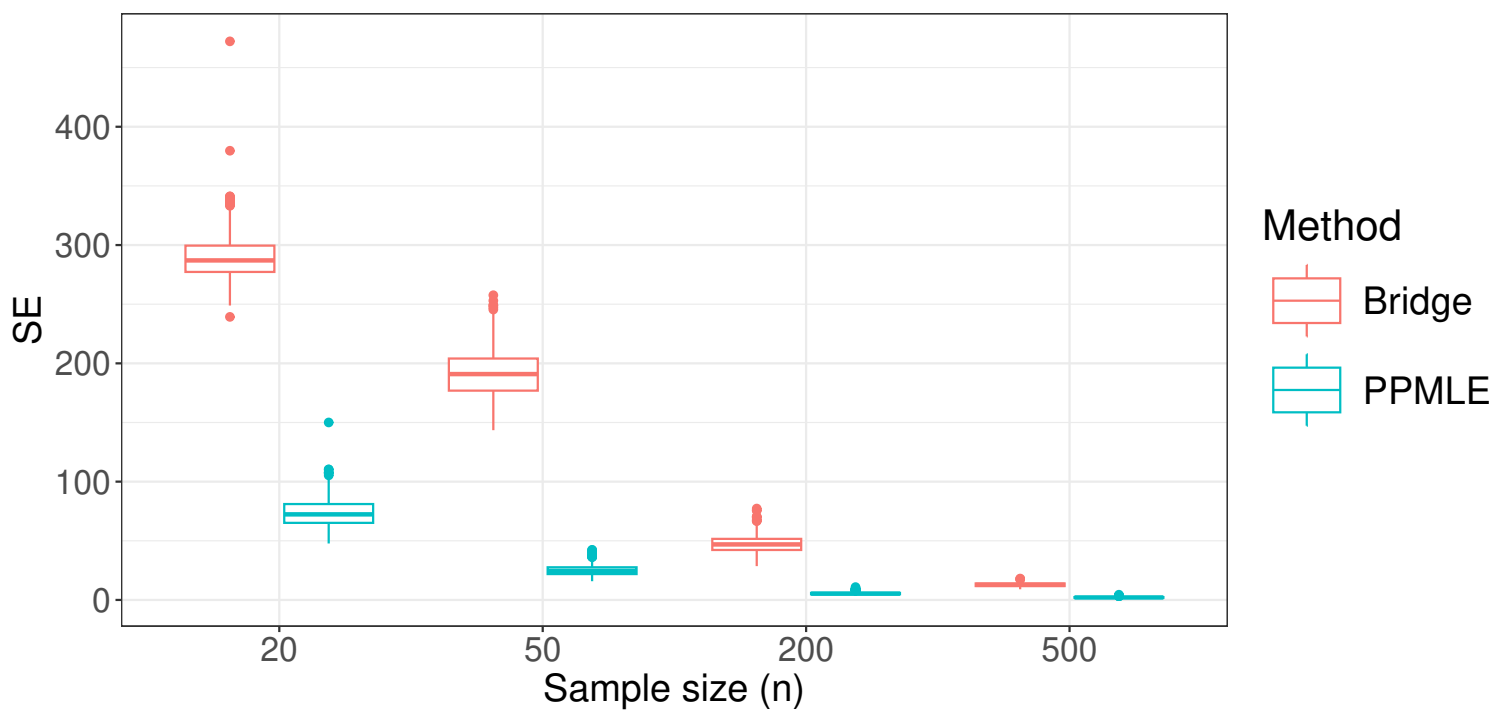

Fig. S10: SE comparison between Σ and $\hat{\Sigma}$ for the PPMLE and the bridge function estimator, for $d = 30$ variables, over $N = 500$ iterations.

Sample size	20	50	200	500
PPMLE	0.72 (0.02)	0.77 (0.02)	0.82 (0.01)	0.84 (0.01)
Bridge	0.68 (0.03)	0.71 (0.02)	0.78 (0.01)	0.82 (0.01)

Table S1: Estimated AUC for $d = 30$ variables computed from $N = 500$ replications, for each method and sample size in the second simulation protocol. The standard errors of each estimate are given in parentheses.

B.2 Comparison with the classical graphical Lasso

In order to investigate the behavior of the classical glasso approach, in particular in the case of rare events binary data, we have generated a simple simulation design,

	AUC on discrete pairs		AUC on CD pairs	
Sample size	PPMLE	Bridge	PPMLE	Bridge
20	0.65 (0.04)	0.62 (0.06)	0.73 (0.09)	0.71 (0.1)
50	0.67 (0.03)	0.64 (0.06)	0.8 (0.07)	0.79 (0.08)
200	0.68 (0.02)	0.62 (0.04)	0.87 (0.05)	0.84 (0.05)
500	0.67 (0.01)	0.60 (0.03)	0.88 (0.04)	0.87 (0.05)

Table S2: Estimated AUC for $d = 30$ variables computed from $N = 500$ replications, for each method, sample size and scenario, for the discrete-discrete and continuous-discrete (CD) pairs of variables. The standard errors of each estimate are given in parentheses.

with a block-diagonal correlation matrix with homogeneous values in each block as presented below.

$$\Sigma = \begin{pmatrix} \underbrace{0.6}_{7\times 7} & 0 & 0 & 0 \\ 0 & \underbrace{0.7}_{10\times 10} & 0 & 0 \\ 0 & 0 & \underbrace{0.8}_{8\times 8} & 0 \\ 0 & 0 & 0 & \underbrace{0.9}_{5\times 5} \end{pmatrix}$$

We considered three types of variables, as in the real data application : binary data with a low probability parameter $\mathcal{B}(0.01)$ to represent mutations, negative binomial with high values $NB(1000, 0.3)$ to illustrate RNA-seq counts and continuous variables $\mathcal{N}(0, 1000)$ for protein expression. Four sample sizes of $150, 200, 300$ and 500 were considered in order to guarantee at least one occurrence of 1 in the binary variables.

Figure S11 represents the ROC curves obtained for each method, averaged over $N = 100$ simulations, according to a penalization parameter $\lambda \in \log(1.01, 1.02, ...3)$. When considering all types of variables, it can be observed that the proposed PPMLE approach performs better than the classical graphical Lasso, with AUC values around 0.9 for PPMLE for all the considered sample sizes, and between 0.81 and 0.89 for the glasso (Table S3). The difference is even larger in the case of binary-discrete data, as shown in Figure S12. This is confirmed by the calculation of the AUC values presented in Table S4, that are around 0.83 for the PPMLE and between 0.61 and 0.73 for the glasso.

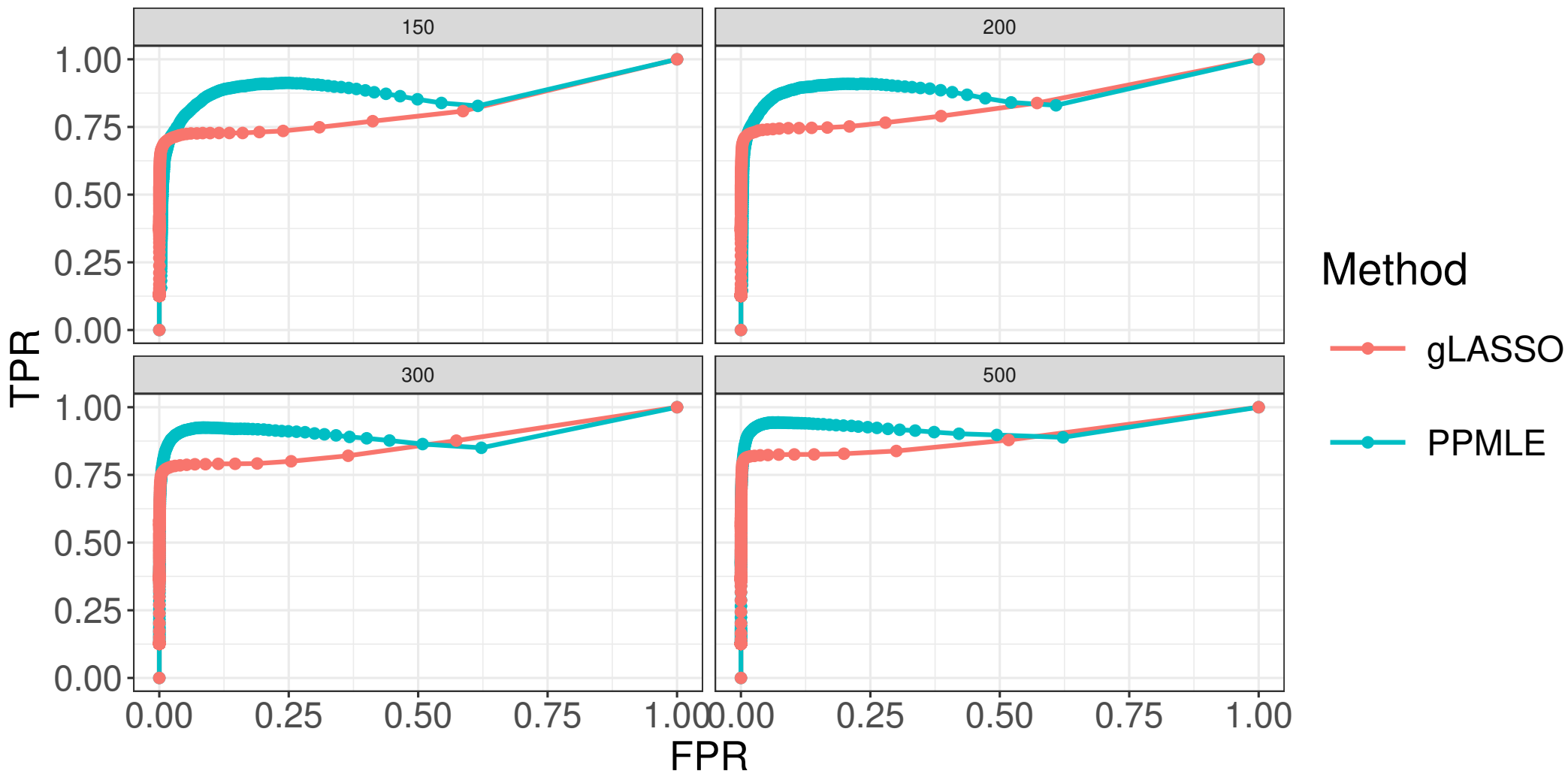

Fig. S11: ROC curves for the PPMLE and gLasso for various sample sizes $n \in \{150, 200, 300, 500\}$, averaged over $N = 100$ replications, for the block matrix simulation scenario.

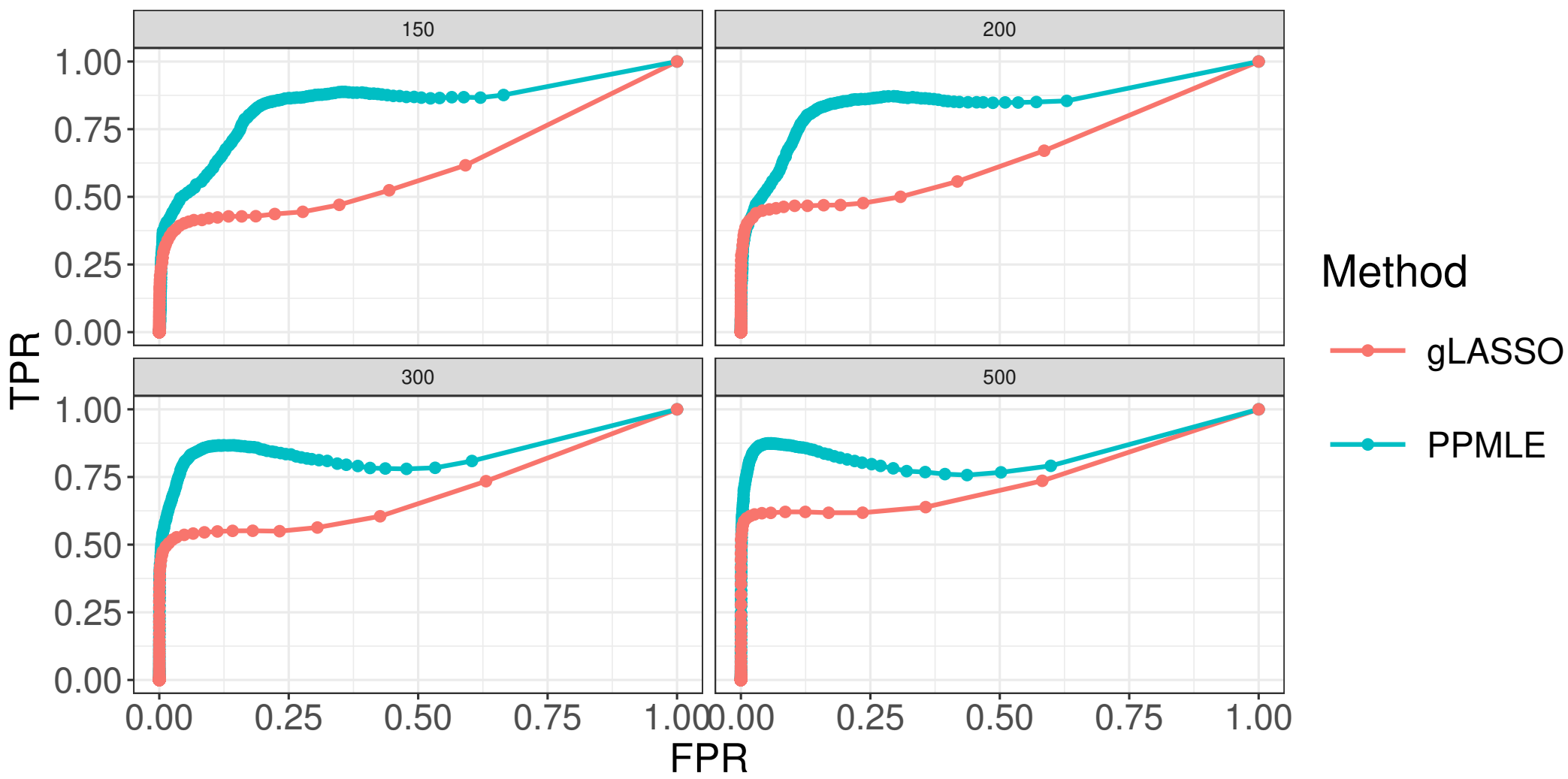

Fig. S12: ROC curves for the PPMLE and gLasso for various sample sizes $n \in \{150, 200, 300, 500\}$, restricted to the coefficients of Σ between binary and discrete variables, averaged over $N = 100$ replications, for the block matrix simulation scenario.

Sample size	150	200	300	500
PPMLE	0.88 (0.03)	0.88 (0.03)	0.90 (0.03)	0.92 (0.02)
gLasso	0.81 (0.03)	0.84 (0.04)	0.87 (0.02)	0.89 (0.02)

Table S3: Estimated AUC for $d = 30$ variables over $N = 100$ replications, for the PPMLE and gLasso for various sample sizes $n \in \{150, 200, 300, 500\}$, for the block matrix simulation scenario. The standard errors of each estimate are given in parentheses.

Sample size	150	200	300	500
PPMLE	0.83 (0.07)	0.84 (0.07)	0.83 (0.05)	0.83 (0.04)
gLasso	0.61 (0.07)	0.65 (0.07)	0.69 (0.06)	0.73 (0.05)

Table S4: Estimated AUC for $d = 30$ variables over $N = 100$ replications, for the PPMLE and gLasso for various sample sizes $n \in \{150, 200, 300, 500\}$, restricted to the coefficients of Σ between binary and discrete variables, for the block matrix simulation scenario. The standard errors of each estimate are given in parentheses.

C Comparison to other methods on real data

The classical graphical Lasso (computed from Pearson's correlation matrix of the observed data) as well as the bridge function estimator have been run on the real data set studied in Section 4. Figures S13 and S14 represent the HBIC as well as the corresponding copula partial correlation network obtained for the bridge functions estimator. The optimal λ HBIC-wise is detected as very low, so the graph is nearly not penalized and almost all possible edges are kept without any selection.

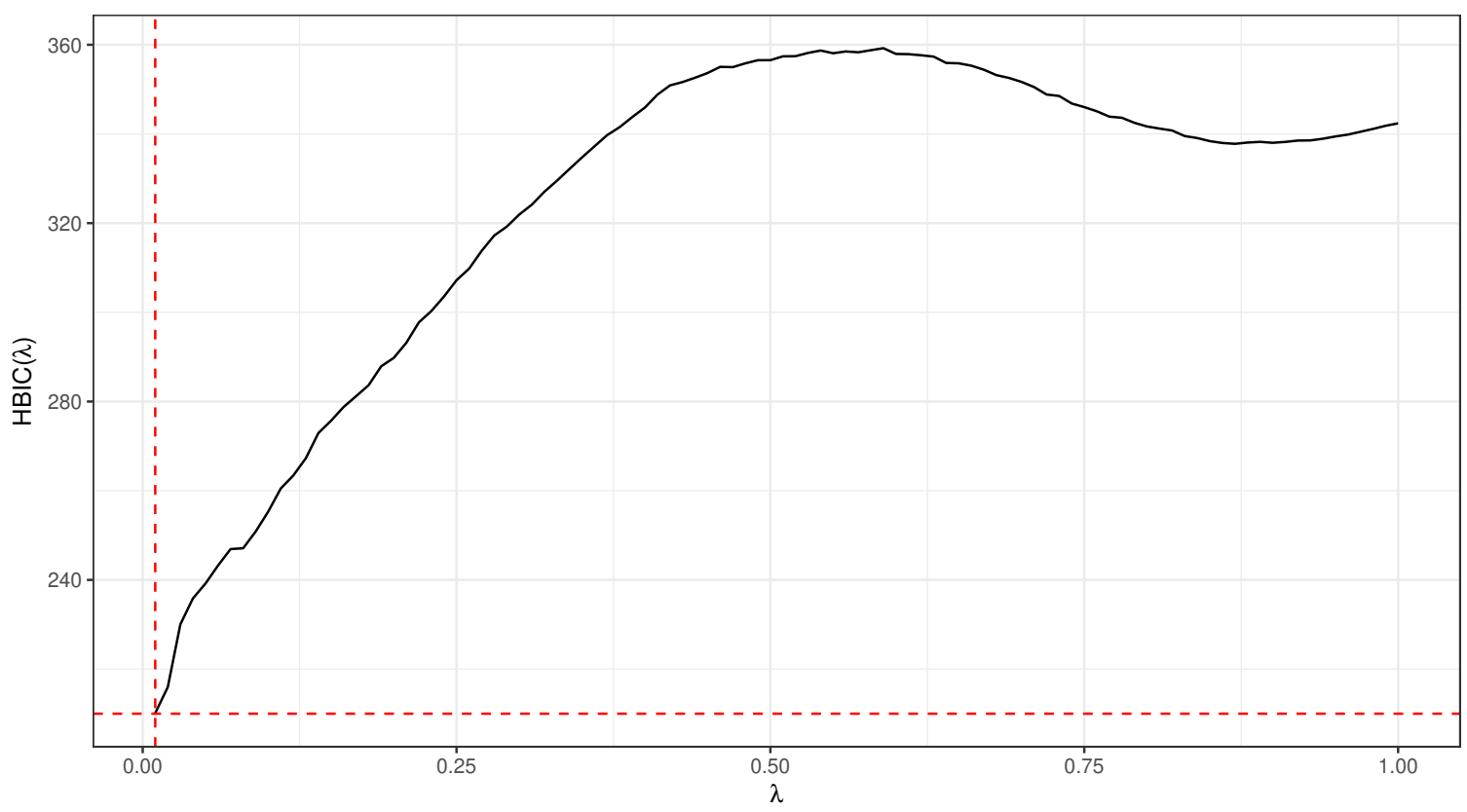

Fig. S13: Values of HBIC(λ), $0.05 < \lambda \leq 0.75$ for the Bridge functions estimation method.

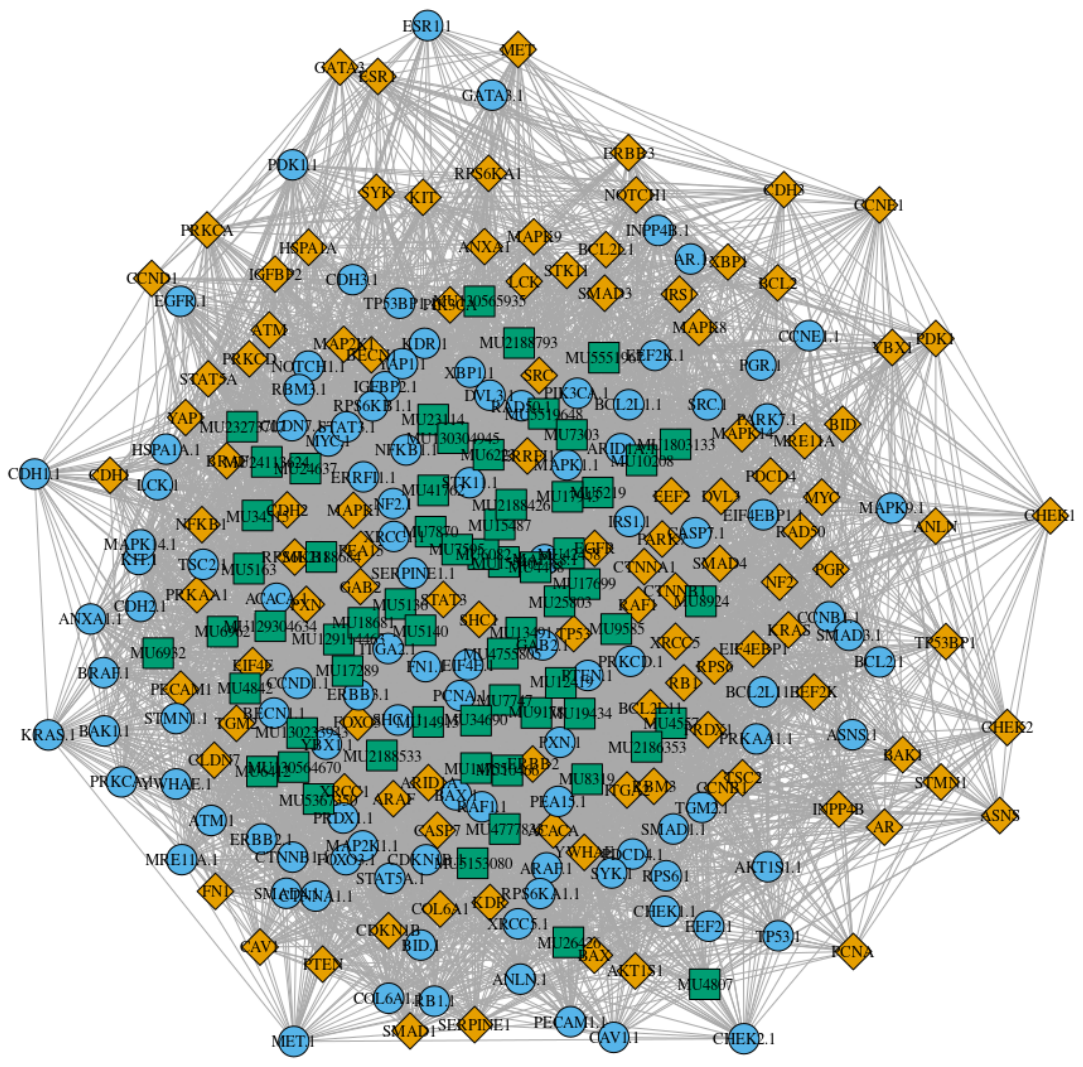

Fig. S14: Copula partial correlation graph for the bridge functions estimator, thresholded at the optimal $\lambda = 0.05$ HBIC-wise.

Figures S15 and S16 represent the HBIC as well as the partial correlation network obtained for the graphical Lasso estimator. The optimal λ and the network structure are similar to the results obtained for the PPMLE in Section 4. However, the clique of mutations is missing from the main subgraph in comparison to the PPMLE. This confirms the results obtained in the simulation study presented above, and highlights the difficulty of the glasso approach to deal with rare events binary data.

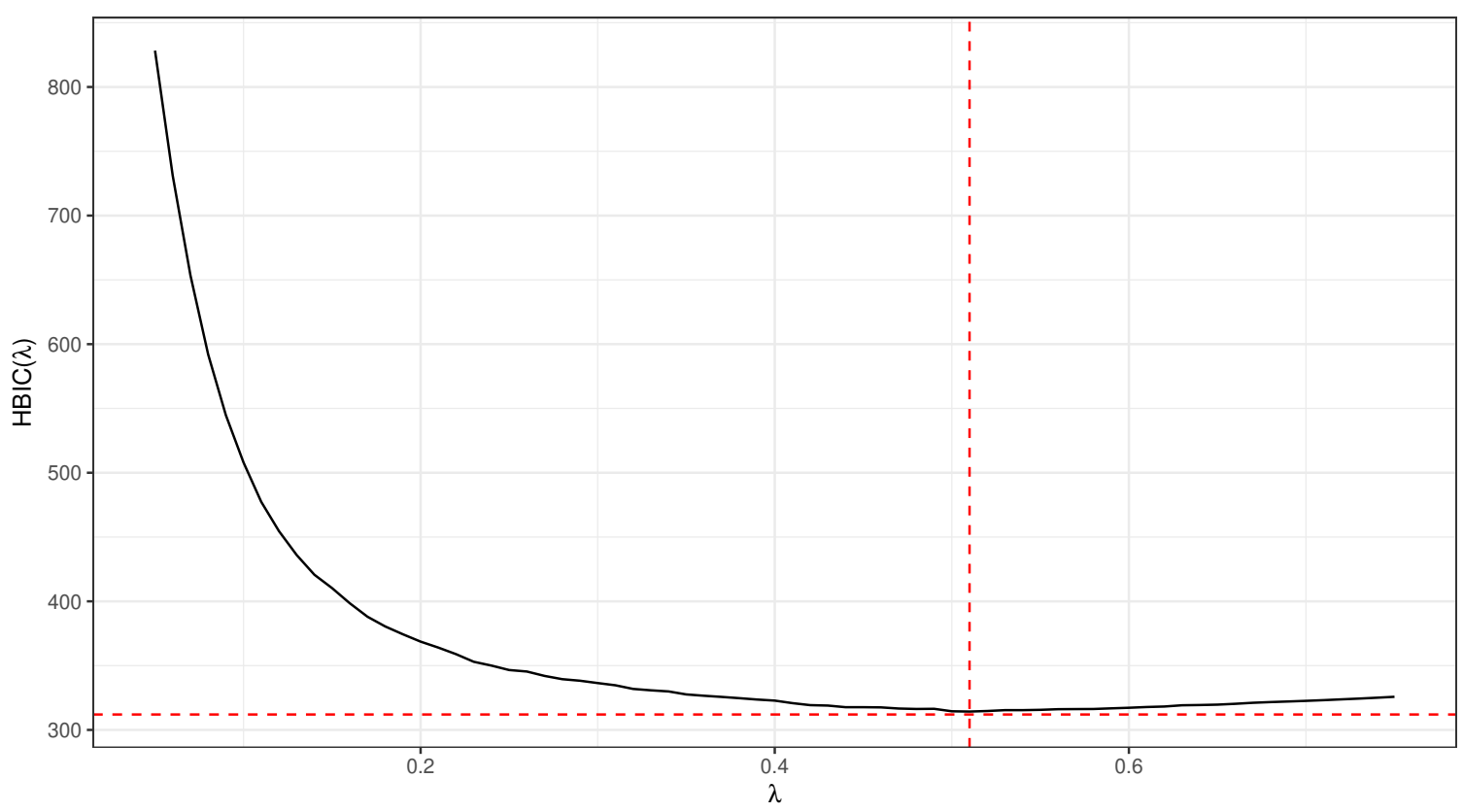

Fig. S15: Values of HBIC(λ), $0.05 < \lambda \leq 0.75$ for the classical graphical lasso.

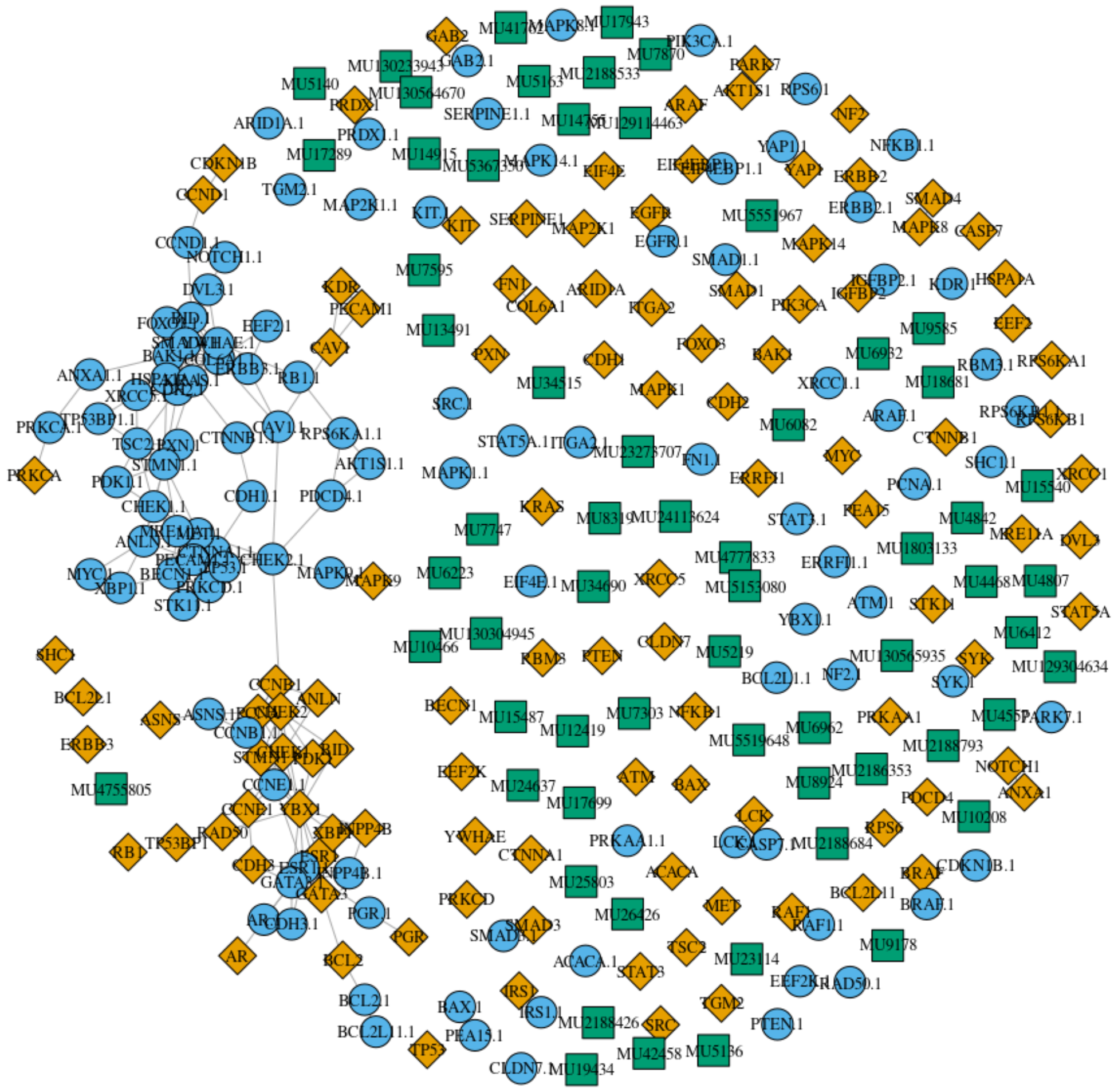

Fig. S16: Partial correlation graph between the RNA-seq counts (yellow diamonds), proteins (blue circles) and mutations (green squares) for the graphical Lasso estimator, thresholded at the optimal $\lambda = 0.51$ HBIC-wise.

D Copula distribution function

Let us show that if $Z^i = (Z_1^i, ..., Z_d^i) \sim \mathcal{N}(0, \Sigma)$ is a centered standardized Gaussian vector of correlation matrix Σ, and $X_j^i = F_j^{\leftarrow}(\Phi(Z_j^i))$ such that $F_j^{\leftarrow}(u) = \inf\{x : F_j(x) \geq u\}$, then X^i has the CDF given by (1). Indeed,

$$\begin{aligned} \mathbb{P}(X_1^i \leq x_1, \dots, X_d^i \leq x_d) &= \mathbb{P}(F_1^{\leftarrow}(\Phi(Z_1^i)) \leq x_1, \dots, F_d^{\leftarrow}(\Phi(Z_d^i)) \leq x_d) \\ &= \mathbb{P}(\Phi(Z_1^i) \leq F_1(x_1), \dots, \Phi(Z_d^i) \leq F_d(x_d)) \end{aligned}$$

$$= \mathbb{P}(Z_1^i \leq \Phi(F_1(x_1)), \dots, Z_d^i \leq \Phi(F_d(x_d)))$$
$$= \Phi_\Sigma(\Phi(F_1(x_1)), \dots, \Phi(F_d(x_d))),$$

The key step, $F_j^{\leftarrow}(\Phi(Z_j)) \leq x_j \iff \Phi(Z_j) \leq F_j(x_j)$, or more generally that $t \leq F(s) \iff F^{\leftarrow}(t) \leq s$ for any (t, s), is shown in [34].

E Simulation of the precision matrix

First, a matrix $\tilde{\Omega}$ is simulated via Algorithm 1. Note that the Watts-Storgatz approach is implemented in the `igraph` package [35], and that the last step is necessary to ensure positive definiteness of $\tilde{\Omega}$.

Algorithm 1 Simulation of $\tilde{\Omega}$ for p variables (Zhang et al. [10])

Require: $p > 0$ the number of variables, $r > 0$ the number of non-null coefficients
  Define a $p \times p$ matrix of zeroes $\tilde{\Omega}$
  Randomly assign $r/2$ non-null coefficients by a Watts-Storgatz approach [19] in the upper triangular part of $\tilde{\Omega}$
  Fill these coefficients with $\mathcal{U}(0.2, 0.7)$ values
  $\tilde{\Omega} \leftarrow \tilde{\Omega} + \tilde{\Omega}^T$
  $\text{diag}(\tilde{\Omega}) \leftarrow 1$
  Compute $\lambda_m = \min(Sp(\tilde{\Omega}))$
  $\text{diag}(\tilde{\Omega}) \leftarrow |\lambda_m| + 0.01$

Then, compute $\tilde{\Sigma} = \tilde{\Omega}^{-1}$. Because we suppose model (1) to be parametrized by a correlation matrix, standardize it to obtain $\Sigma = \Lambda^{-\frac{1}{2}}\tilde{\Sigma}\Lambda^{-\frac{1}{2}}$ where $\Lambda = \text{diag}(\tilde{\Sigma})$. Finally, compute our matrix of interest $\Omega = \Sigma^{-1} = \Lambda^{\frac{1}{2}}\tilde{\Omega}\Lambda^{\frac{1}{2}}$.

F Details of estimation methods

F.1 Estimation based on bridge functions

A first frequentist approach in order to estimate Ω has been given by Liu et al. in the nonparanormal SKEPTIC [17] and consists in the following steps:

1. Estimate Kendall's τ on the observed variables
2. Find the root of the corresponding bridge function in order to obtain the covariance matrix Σ between the latent Gaussian variables
3. Invert Σ in order to obtain Ω as described in section 2.2

Note that the difference with our estimation method comes from the additional step while we directly estimate Σ on the observed variables by PPMLE before inversion.

A theoretical expression for Kendall's tau between two variables X_j and X_k is given by:

$$\tau_{jk} = \text{Corr}\left(\text{sign}(X_j - \tilde{X}_j)\text{sign}(X_k - \tilde{X}_k)\right) \tag{10}$$

where $\tilde{X}_j$ and $\tilde{X}_k$ denote two independent copies of X_j and X_k. It can be estimated by

$$\hat{\tau}_{jk} = \frac{2}{n(n-1)} \sum_{1 \le i < i' \le n} \text{sign}(x_j^i - x_j^{i'})\text{sign}(x_k^i - x_k^{i'}) \tag{11}$$

Moreover, when both variables are continuous, we have the following result [36]:

$$\Sigma_{jk} = 2\sin\left(\frac{\pi}{2}\tau_{jk}\right) \tag{12}$$

and it is sufficient to plug the estimator from equation (11) into equation (12) in order to estimate $\hat{\Sigma}_{jk}$.

If at least one of the variables is binary, the estimator from equation (11) holds because $\text{sign}(x_j^i - x_j^{i'}) = x_j^i - x_j^{i'}$. However, the bridge function becomes [18]:

$$\begin{aligned} \tau_{jk} &= B^{(bc)}(\Sigma_{jk}, \Delta_j) \\ &= 4(\Phi_{\Sigma_{jk}/\sqrt{2}}(\Delta_j, 0)) - 2\Phi(\Delta_j) \end{aligned} \tag{13}$$

where Δ_j denotes the cutoff value for a latent variable Z_j behind the binary variable X_j. In practice, one can estimate Δ_j by $\hat{\Delta}_j = \Phi^{-1}(1 - \bar{X}_j)$ and $\hat{\Sigma}_{jk}$ by solving $B(t, \hat{\Delta}_j) - \hat{\tau}_{jk} = 0$. Similarly, in the case where both variables are binary, the bridge function becomes

$$\begin{aligned} \tau_{jk} &= B^{(bb)}(\Sigma_{jk}, \Delta_j, \Delta_k) \\ &= 2\Phi_{\Sigma_{jk}}(\Delta_j, \Delta_k) - 2\Phi(\Delta_j)\Phi(\Delta_k) \end{aligned} \tag{14}$$

Finally, these expressions have been extended to the case of discrete variables with more than two modalities [10]. In place of Kendall's τ, a new rank statistic r_{jk} is introduced for the discrete-discrete and mixed cases in order to facilitate computation of the bridge functions. When both variables are multinomial, we compute $\hat{r}_{jk}$ on the observed data as below:

$$\hat{r}_{jk} = \frac{2}{n(n-1)} \sum_{1 \le i < i' \le n} (x_j^i - x_j^{i'})(x_k^i - x_k^{i'}) \tag{15}$$

We also get the associated bridge function

$$\begin{aligned} r_{jk} &= \mathbb{E}(\hat{r}_{jk}) \\ &= B^{(dd)}(\Sigma_{jk}, \Delta_j, \Delta_k) \\ &= 2\{\sum_{l=1}^{L} \sum_{m=1}^{L} \Phi_{\Sigma_{jk}}(\Delta_{jl}, \Delta_{km}) - \sum_{l=1}^{L} \Phi(\Delta_{jl}) \sum_{m=1}^{L} \Phi(\Delta_{km})\} \end{aligned} \tag{16}$$

When X_j is discrete and X_k is continuous, we compute:

$$\hat{r}_{jk} = \frac{2}{n(n-1)} \sum_{1\leq i<i'\leq n} (x_j^i - x_j^{i'})\text{sign}(x_k^i - x_k^{i'}) \tag{17}$$

The associated bridge function is given by:

$$\begin{aligned} r_{jk} &= \mathbb{E}(\hat{r}_{jk}) \\ &= B^{(dc)}(\Sigma_{jk}, \Delta_j, \Delta_k) \\ &= 2\{\sum_{l=1}^{L} \Phi_{\Sigma_{jk}/\sqrt{2}}(\Delta_{jl}, 0) - 2\sum_{l=1}^{L} \Phi(\Delta_{jl})\} \end{aligned} \tag{18}$$

The main limit of this method is the lack of roots for the bridge functions when the sample size n is not large enough, and the needed adaptation of the code as in https://github.com/Aiying0512/LGCM. Also, the case when a binary variable takes the same modality for all observations is not taken into consideration.

F.2 Pairwise densities

The densities $\hat{f}_{jj'}$ introduced in section 2.2 take the form below depending on the case. For easier notation, let $\hat{F}_j(x_j) = u$, $\hat{F}_{j'}(x_{j'}) = v$, $\hat{F}_j(x_j-) = u-$ and $\hat{F}_{j'}(x_{j'}-) = v-$, where x_j- and $x_{j'}-$ denote the points before x_j and $x_{j'}$ in the supports of $\hat{F}_j$ and $\hat{F}_{j'}$. If both variables are continuous, we have

$$\hat{f}_{jj'}(x_j, x_{j'}, \Sigma_{jj'}) = \frac{1}{\sqrt{2\pi(1-\Sigma_{jj'}^2)}} \exp\left(-\frac{\Phi^{-1}(u)^2 + \Phi^{-1}(v)^2 - 2\Sigma_{jj'}\Phi^{-1}(u)\Phi^{-1}(v)}{2(1-\Sigma_{jj'}^2)}\right)$$

If X_j is continuous and $X_{j'}$ is discrete, we have

$$\hat{f}_{jj'}(x_j, x_{j'}, \Sigma_{jj'}) = \Phi\left(\frac{\Phi^{-1}(v) - \Sigma_{jj'}\Phi^{-1}(u)}{1-\Sigma_{jj'}^2}\right) - \Phi\left(\frac{\Phi^{-1}(v-) - \Sigma_{jj'}\Phi^{-1}(u)}{1-\Sigma_{jj'}^2}\right)$$

If both variables are discrete, we have

$$\hat{f}_{jj'}(x_j, x_{j'}, \Sigma_{jj'}) = \int_{u-}^{u} \Phi\left(\frac{\Phi^{-1}(v) - \Sigma_{jj'}\Phi^{-1}(z)}{1-\Sigma_{jj'}^2}\right) - \Phi\left(\frac{\Phi^{-1}(v-) - \Sigma_{jj'}\Phi^{-1}(z)}{1-\Sigma_{jj'}^2}\right) dz$$

F.3 Graphical Lasso

Suppose that $\mathbf{Z} = (Z_1, ..., Z_d) \sim \mathcal{N}(0, \Sigma)$ is a centered standardized Gaussian vector of correlation matrix Σ. Its density is expressed as, for $\mathbf{z} = (z_1, \ldots, z_d)$:

$$f(\mathbf{z}) = \frac{1}{\sqrt{(2\pi)^d |\Sigma|}} \exp\left(-\frac{\mathbf{z}^T \Sigma^{-1} \mathbf{z}}{2}\right).$$

Let $\hat{\Sigma}^P$ denote the estimator of the Pearson correlation matrix with elements:

$$(\hat{\Sigma}^P)_{jk} = \frac{1}{n} \sum_{i=1}^{n} Z_j^i Z_k^i.$$

A natural log-MLE estimator of $\Omega = \Sigma^{-1}$, for n independent realizations $Z_1^i, \ldots, Z_d^i$, $i = 1, \ldots, n$ is:

$$\begin{aligned}
\hat{\Omega} &= \underset{\Omega}{\text{argmax}} \frac{1}{n} \sum_{i=1}^{n} \log\left(\frac{1}{\sqrt{(2\pi)^d |\Omega^{-1}|}} \exp\left(-\frac{(Z_1^i, \ldots, Z_d^i)^T \Omega (Z_1^i, \ldots, Z_d^i)}{2}\right)\right) \\
&= \underset{\Omega}{\text{argmax}} \left(\frac{1}{n} \sum_{i=1}^{n} \log|\Omega| - \frac{1}{2}(z_1^i, \ldots, z_d^i)^T \Omega (z_1^i, \ldots, z_d^i)\right) \\
&= \underset{\Omega}{\text{argmax}} \left(\frac{1}{n} \sum_{i=1}^{n} \log|\Omega| - \sum_{j=1}^{d} \sum_{k=1}^{d} \frac{z_j^i z_k^i \Omega_{jk}}{2}\right) \\
&= \underset{\Omega}{\text{argmax}} \left(\log|\Omega| - \sum_{j=1}^{d} \sum_{k=1}^{d} \Omega_{jk} \frac{1}{n} \sum_{i=1}^{n} z_j^i z_k^i\right) \\
&= \underset{\Omega}{\text{argmax}} \left(\log|\Omega| - \sum_{j=1}^{d} \sum_{k=1}^{d} \Omega_{jk} \hat{\Sigma}_{kj}^P\right) \\
&= \underset{\Omega}{\text{argmax}} \left(\log|\Omega| - tr(\Omega \hat{\Sigma}^P)\right).
\end{aligned}$$

Equation (4) is then obtained by penalizing the above likelihood and substituting our estimator (3) for $\hat{\Sigma}^P$.
